# Genome Expression Profile Analysis of the Immature Maize Embryo during Dedifferentiation

**DOI:** 10.1371/journal.pone.0032237

**Published:** 2012-03-20

**Authors:** Yaou Shen, Zhou Jiang, Xiadong Yao, Zhiming Zhang, Haijian Lin, Maojun Zhao, Hailan Liu, Huanwei Peng, Shujun Li, Guangtang Pan

**Affiliations:** 1 Key Laboratory of Biology and Genetic Improvement of Maize in Southwest Region, Maize Research Institute of Sichuan Agricultural University, Ministry of Agriculture, Wenjiang, People's Republic of China; 2 College of Life and Science, Sichuan Agricultural University, Ya'an, People's Republic of China; 3 Institute of Animal Nutrition, Sichuan Agricultural University, Ya'an, People's Republic of China; University of Arizona, United States of America

## Abstract

Maize is one of the most important cereal crops worldwide and one of the primary targets of genetic manipulation, which provides an excellent way to promote its production. However, the obvious difference of the dedifferentiation frequency of immature maize embryo among various genotypes indicates that its genetic transformation is dependence on genotype and immature embryo-derived undifferentiated cells. To identify important genes and metabolic pathways involved in forming of embryo-derived embryonic calli, in this study, DGE (differential gene expression) analysis was performed on stages I, II, and III of maize inbred line 18-599R and corresponding control during the process of immature embryo dedifferentiation. A total of ∼21 million cDNA tags were sequenced, and 4,849,453, 5,076,030, 4,931,339, and 5,130,573 clean tags were obtained in the libraries of the samples and the control, respectively. In comparison with the control, 251, 324 and 313 differentially expressed genes (DEGs) were identified in the three stages with more than five folds, respectively. Interestingly, it is revealed that all the DEGs are related to metabolism, cellular process, and signaling and information storage and processing functions. Particularly, the genes involved in amino acid and carbohydrate transport and metabolism, cell wall/membrane/envelope biogenesis and signal transduction mechanism have been significantly changed during the dedifferentiation. To our best knowledge, this study is the first genome-wide effort to investigate the transcriptional changes in dedifferentiation immature maize embryos and the identified DEGs can serve as a basis for further functional characterization.

## Introduction

Maize is a major commodity in international agriculture and an important source of protein and energy for human and livestock. It has been one of the prime targets of genetic manipulation. However, the genetic transformation of maize still greatly depends on immature embryo-derived undifferentiated cells (called embryonic callus) [1] and is strongly genotype-dependent, because there is obvious difference of the dedifferentiation frequency for immature embryo among various genotypes. In particular, some of the maize inbred lines even fail to induct embryonic calli [2], [3], [4], [5]. Previous studies have revealed that the dedifferentiation efficiency is a quantitative trait controlled by the additive genes effect with the hereditary capacity of more than 90% [6].

With the described method of composite interval mapping, five quantitative trait locis (QTLs) have been identified on chromosome 1, 3, 7 and 8, respectively, to be responsible for dedifferentiation efficiency, which accounts for 5.25∼23.4% phenotypic variation [7]. Currently, however, many genes involved in dedifferentiation have not been isolated and the molecular mechanism of maize embryogenic callus induction is still poorly understood. Complement of Maize Genome Project makes it possible to detect functional genes on a genome-wide scale. Previous study indicated that maize inbred line 18-599R [8], [9] is an elite line with high introduction efficiency of embryonic callus (more than 90%) compared to other lines. Therefore, in this study, we expect to reveal important genes involved in the form of embryo-derived embryonic calli by detecting differentially expressed genes in 18-599R during the process of embryo dedifferentiation using DGE (differential gene expression) technologies. The study will help to elucidate the mechanism of immature embryo dedifferentiation, and provide important evidence for breeding excellent transgenic acceptor line with high introduction efficiency of embryonic callus. In comparison with the control, 251, 324 and 313 differentially expressed genes (DEGs) were identified in the stages I, II, and III, respectively. Interestingly, it is revealed that all the DEGs are related to metabolism, cellular process, and signaling and information storage and processing functions. Particularly, the genes involved in amino acid and carbohydrate transport and metabolism, cell wall/membrane/envelope biogenesis and signal transduction mechanism have been significantly changed during the dedifferentiation.

## Materials and Methods

### Samples and RNA isolation

According to transformation of morphological feature, dedifferentiation of immature embryo for inbred line 18-599R [8], [9] can be divided to three stages (Figure S1): stage I (embryo intumesces, 1–5 d after inoculation), stage II (initial callus forms, 6–10 d after inoculation) and stage III (embryonic callus forms, 11–15 d after inoculation). Line 18-599R was grown in a growth chamber with a photoperiod of 14 h light/10 h dark at 27°C with the relative humidity of 70%. Immature ear was harvested from plants at 12 d after self-pollination, and immature embryo (1.5–1.8 mm) was isolated and laid on modified N6 inducting medium under aseptic conditions (Table S1), and subjected to aphotic culture at 27°C for 15 days. Total RNA was isolated from each sample from a pool of 10 calli at 0–15 d after inoculating embryo on medium using Trizol (Invitrogen). 0 d RNA sample (separated from immature ear but not cultured on N6 medium) was used as control (immature embryo). RNA samples from 1–5 d (stage I sample) were mixed with equal proportion, as well as samples from 6–10 d (stage II sample) and 11–15 d (stage III sample). Then, the three samples, along with the control sample, were separately submitted to digital gene expression profiling (DGE) based on Solexa sequencing.

### Library construction and Solexa sequencing

20 ug of total RNA and 6 ug of mRNA were purified by adsorption of biotin oligo magnetic beads. After mRNA's binding, cDNA synthesis was performed. Double strand cDNA was introduced into cDNA fragment digested by *NlaIII* endonuclease and these binging fragment containing sequences of CATG site and adjacent poly A tail in 3′ end. After the precipitation of 3′ cDNA fragment, Illumina adaptor 1 was added to 5′ end. Both adaptor 1 and CATG site can be recognized by *MmeI*, which cut at downstream CATG site and produce fragment of 17 bp tags with adaptor 1. Adaptor 2 was added to the 3′ end of these tags after getting rid of fragment with beads in 3′ end. Then these sequences were prepared for Solexa sequencing.

### Sequence annotation

Clean-tags were obtained by filtering the adaptor sequences and removing low-quality sequences (containing ambiguous bases). Then, the clean tags were mapped to the reference genome and genes of maize available at ftp://ftp.maizesequence.org/pub/maize/release-5b. Only the tags with perfect match or one mismatch were further considered and annotated based on the reference genes. The expression level of each gene was estimated by the frequency of clean tags and then normalized to TPM (number of transcripts per million clean tags) [10], which is a standard method and extensively used in DGE analysis [11]. TPM indicates reads per kilobase of transcript per million of sequenced reads. The expression level of each gene was measured by the normalized number of matched clean tags.

KOG functional classification, Gene Ontology (GO) and pathway annotation and enrichment analyses were based on the NCBI COG (http://www.ncbi.nlm.nih.gov/COG) [12], Gene Ontology Database (http://www.geneontology.org/) [13] and KEGG pathway (http://www.genome.jp/kegg/) [14], respectively.

### Identification of different expression genes

The probability that one gene G is equally expressed in two samples can be illustrated by the following formula [15]:
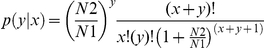



N1 and N2 denotes the total number of clean tags in two compared libraries, respectively, while x and y represents the clean tags mapping to gene G. P value indicates the significance of prospect differences of transcript accumulation. The threshold of P value in multiple test and analysis was determined by FDR (False Discovery Rate) [16]. A combination of FDR<0.001 and the absolute value of log2-Ratio> = 1 were used as the threshold to determine the significance of gene expression difference.

### GO and pathway enrichment analysis of DEGs

GO and pathway enrichment analysis was used to identify the significantly enriched functional classification or metabolic pathways in DEGs. The formula is [17]:
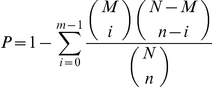
N is the total number of genes with GO/KEGG functional annotations, and n is the number of DEGs in N. M is number of the gene with specific GO/KEGG annotations, and m is the number of DEGs in M.

### Real-time PCR

To validate the DGEs obtained from Solexa sequencing, 12 genes (GRMZM2G016890, GRMZM2G071272, GRMZM2G112377, GRMZM2G458401, GRMZM2G474755, GRMZM2G086066, GRMZM2G150276, GRMZM2G156877, GRMZM2G008247, GRMZM2G011789, GRMZM2G170692, GRMZM2G011789) were subjected to quantitative real-time PCR analysis using Bio-Rad CFX96. Actin1 (GRMZM2G126010) was used as the endogenous control. cDNA synthesis was carried out using 1 µg total RNA. The corresponding primers were designed using Beacon Designer 7 software and listed in (Table S2.) The amplification programs were performed according to the standard protocol of the Bio-Rad CFX96 system: 98°C for 2 min; 98°C for 2 s, 59°C for 10 s, 40 cycles, and followed by a thermal denaturing step to generate the melt curves for verification of amplification specificity. All reactions were run in triplicate, including non-template controls. Statistical analysis was performed using the 2^−ΔΔCT^ method.

## Results and Discussion

### Tag identification and saturation analysis

With Solexa sequencing. the total number of sequenced tags for the control and stages I, II and III sample was 5,120,122, 5,403,404, 5,194,757, and 5,394,960, respectively. After filtering the adaptor sequences and removing the low-quality tags, 4,849,453, 5,076,030, 4,931,339, and 5,130,573 clean tags were left (Table 1). Considering of the robustness of subsequently data analysis, only the tag with less than one copy was remained in the four libraries and used for further analysis. Finally, we have obtained 244,759, 271,531, 267,622, and 257,709 unique tags for the control and stages I, II and III, respectively.

**Table 1:** Basic statistics of tags in three stage and control samples.

	Control	Stage I	Stage II	Stage III
Total tag	5120122	5403404	5194757	5394960
Clean tag	4849453	5076030	4931339	5130573
Unique tag	244759	271531	267622	257709
Unique tag copy number >2	130212	118312	114602	115310
Unique tag copy number >5	55063	54420	53282	52206
Unique tag copy number >10	37316	37904	37271	36763
Unique tag copy number >20	24851	25597	25308	25337
Unique tag copy number >50	13480	13844	13827	13936
Unique tag copy number >100	7609	7786	7811	7822

**Figure 1:**
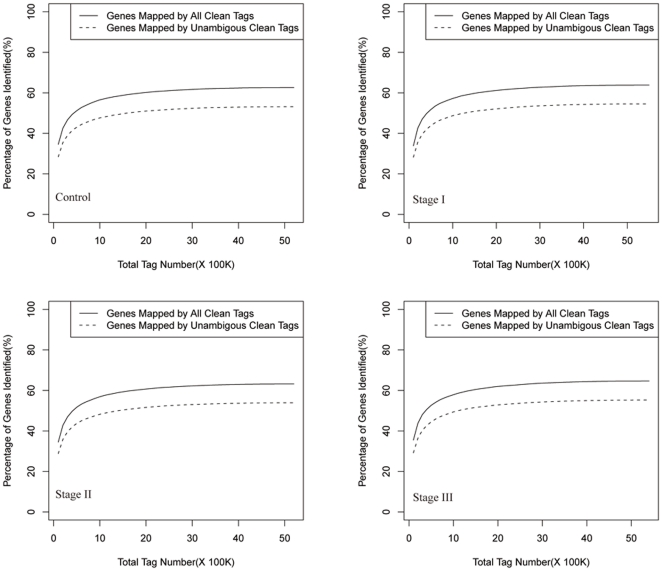
Accumulation the genes mapped by all clean tags (solid line) and unique clean tags (broken line) in four libraries. B1 to B4 denote control, stage I, II and III samples, respectively. Percentage of gene identified (y axis) increases as the total tag number (x axis) increase.

Sequencing data saturation refers to the status that no more new unique tags can be detected with the increases of the number of total tags. In this study, it is shown that all the four libraries can be full representations of the transcripts under the four experiment conditions (Figure 1). Briefly, fewer tags were identified as the number of sequencing tags increases. Then it reached a plateau shortly after 1 M tags were sequenced and no new unique tag was identified as the total number approaching 2 M.

### Annotation of unique tag

A primary step to annotate the tag is to map of clean tags to the reference database [18], [19]. In this study, we used Blastn to map the unique tags against the reference genome and gene sequences of maize, respectively. Only the clean tags that matched perfectly or with one mismatch were analyzed further. Using this criteria, 72,078 (55.35% of unique tags), 67,308 (56.89% of unique tags), 65,267 (56.95% of unique tags) and 66,464 (57.64% of unique tags) in control, stage I, stage II and stage III libraries were mapped to 21,452 (65.93%), 21,729 (66.78%) 21,549 (66.22%) and 22,047 (67.75%) of the reference genes, respectively (Table 2). Meanwhile, 18,951 (14.55%), 18,471 (15.61%), 18,140 (15.83%) and 17,870 (15.5%) unique tags were matched the reference genome, respectively. Among them, 64,158 (49.27%), 60,513 (51.15%), 58,569 (51.11%) and 59,503 (51.6%) unique tags matched to only one gene sequence in the maize genome (Table 2). The large proportion of non-matched clean tags revealed that the efficiency of annotation was low when the copy number was between 2 and 5, which is in accordance with the studies of the transcriptomes of zebrafish [18]. The relative higher mapping efficiency of three culturing stages of immature embryo compared to the control library indicates that more transcripts have been expressed in immature embryo samples during dedifferentiation.

**Table 2:** Statistics of tag mapping against reference gene and genome sequence of maize.

	Control		Stage I		Stage II		Stage III	
	match to genome	match to gene	match to genome	match to gene	match to genome	match to gene	match to genome	match to gene
Unique tag	18951(14.55%)	72078(55.35%)	18471(15.61%)	67308(56.89%)	18140(15.83%)	65267(56.95%)	17870(15.5%)	66464(57.64%)
matched genes		21452(65.93%)		21729(66.78%)		21549(66.22%)		22047(67.75%)
Unique tag matchedto one gene		64158(49.27%)		60513(51.15%)		58569(51.11%)		59503(51.6%)
matched genes		18359(56.42%)		18682(57.41%)		18501(56.86%)		18946(58.22%)

### Comparison and analysis of differentially expressed genes

**Figure 2:**
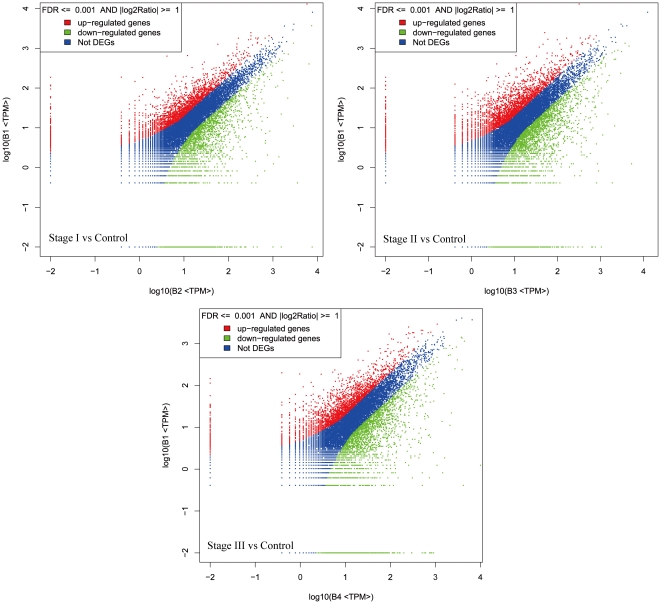
Comparison of gene expression between different libraries. B1 to B4 denote control, stage I, II and III samples, respectively. Blue dots represent the transcripts with no significant expression. Red dots and green dots represent transcripts more abundant in the stage sample and control, respectively. “FDR<0.001” and “absolute value of log2 Ratio≥1” were used as the thresholds to judge the significance of gene expression difference.

**Figure 3:**
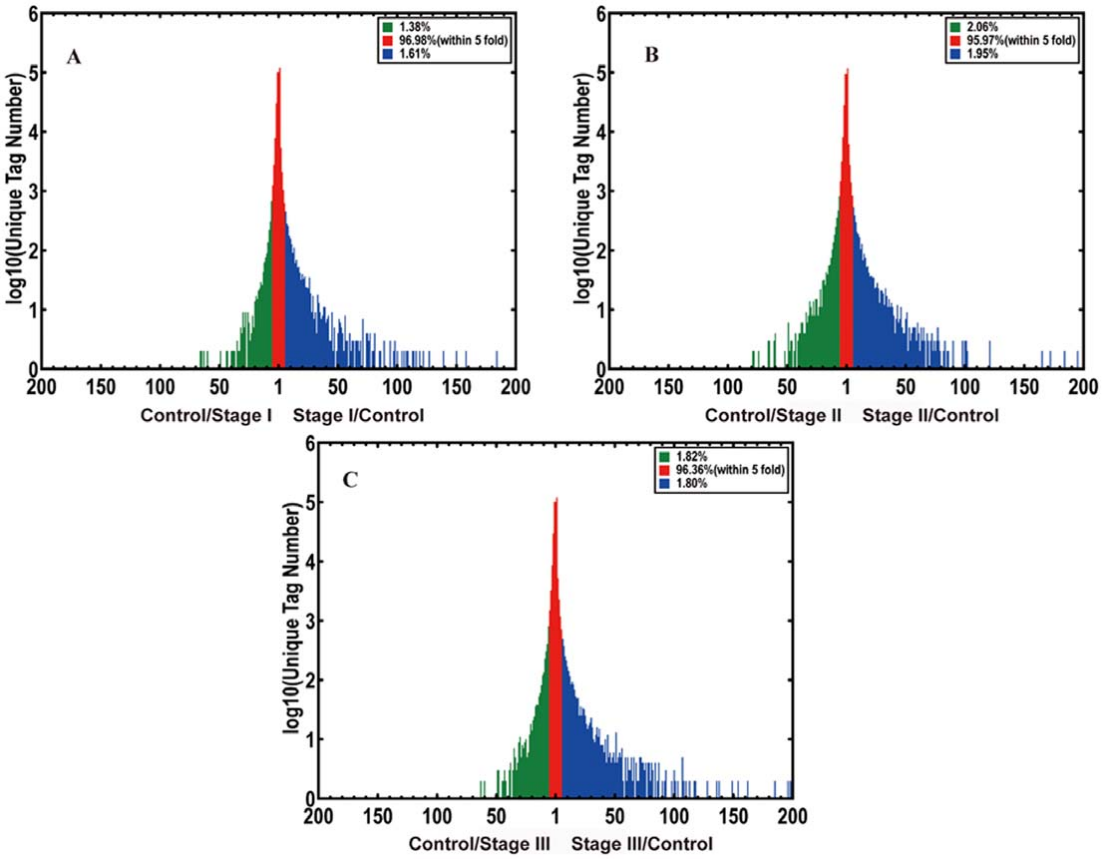
Tags with different expression in stage samples compared to control sample. B1 to B4 denote control, stage I, II and III samples, respectively. Red region represents the differentially expressed tags with differentia expression less than 5 folds. Blue and green region represent the up- and down-regulated tags for more than 5 folds, respectively.

After mapping the tags against the reference genes of maize, the count of the tag corresponding to each gene was calculated in each of the four libraries, which has been used to estimate the gene expression level and the different folds between different samples. The transcripts detected with at least two-fold differences (FDR<0.001 and absolute values of log2 (Ratio) > =  1) in the three immature embryo libraries compare to the control sample are shown in Figure 2. The statistic difference of accumulation of unique tags between them was shown in Figure 3. Among the sample from stage I to stage III, about 1.61%, 1.95%, 1.80% of total unique tags have been increased with at least five-fold, and 1.38%, 2.06%, and 1.82% have been decreased with at least five-fold in the libraries, while the expression levels of the rest unique tags were within five-fold differences.

To investigate the dynamic changes of gene expression, the gene expression level of stage I, stage II and stage III samples was compared with the control, respectively. In comparison with the control, 4,825, 5,119 and 5,463 differentially expressed genes (DEGs) were identified in the three stages, respectively. Among the DEGs (differentially expressed genes) identified with expression differences greater than five folds, 199, 230 and 250 genes were up-regulated in stage I, stage II and stage III sample, respectively. In contrast, the down-regulated DEGs were less abundant in three samples, with only 52, 94, and 63 genes showing different expressions (Table S3, S4 and S5). It is found that the number of up-regulated DEGs among the three stage samples is increased in the progress of dedifferentiation of maize immature embryo, indicating the more genes have been significantly expressed during the progress of dedifferentiation of maize immature embryo. COG Functional annotation of DEGs indicated that both the up-regulated genes and down-regulated ones can be classified into there categories: cellular processes and signaling, metabolism, and information storage and processing. The highest DEGs related to down-regulated expression were the following two: inorganic phosphate transporter 1–6 gene (GRMZM2G112377, 14.19 fold), nuclear transcription factor Y subunit B-6 gene (GRMZM2G124663, 12.47 fold); the up-regulated three were: beta-glucosidase, chloroplastic (GRMZM2G016890, 15.43 fold), dihydroflavonol-4-reductase gene (GRMZM2G099420, 16.68 fold) and oryzain alpha chain gene (GRMZM2G150276, 16.27 fold).

**Figure 4:**
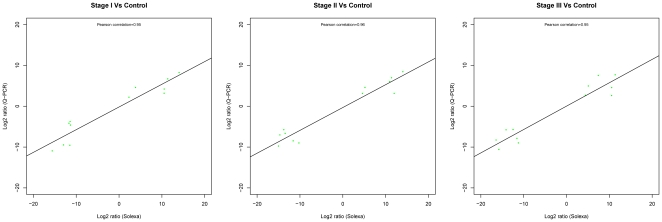
Correlations of the differential expression ratios between Q-PCR and Solexa sequencing in three stages.

To validate the DEGs by DGE based on deep sequencing, 12 genes (GRMZM2G016890, GRMZM2G071272, GRMZM2G112377, GRMZM2G458401, GRMZM2G474755, GRMZM2G086066, GRMZM2G150276, GRMZM2G156877, GRMZM2G008247, GRMZM2G011789, GRMZM2G170692 and GRMZM2G011789) were randomly selected to subject to quantitative real-time PCR analysis. Similarity, expression level in stage I, stage II and stage III samples was compared with the control. As shown in Figure 4, a strong correlation (all Pearson's correlation more than 0.95) was revealed between the DGE data and the quantitative real-time PCR analysis, indicated a good concordance of both methods.

### Pathway enrichment analysis of DEGs

To shed more lights into the functional roles of DEGS responsible for the dedifferentiation of immature embryo, Biological metabolic pathways were investigated by the enrichment analysis of DEGs among the three different stage samples. In stage I, it is revealed that 116 metabolic pathways were affected by up-regulated DEGs (Table S6) and 114 were affected by down-regulated DEGs (Table S7). Fatty acid biosynthesis, metabolism of xenobiotics by cytochrome, alpha-Linolenic acid metabolism pathways were the most significant pathways and were affected by both up- and down- regulated DEGs in stage I sample. In stage II, up- and down-regulated DEGs affected 115 and 116 metabolic pathways, respectively (Table S8 and S9), among which pyruvate metabolism, biosynthesis of plant hormones, fatty acid biosynthesis were three of the most affected pathways. In stage III sample, 117 and 112 pathways were affected by up- and down- regulated DEGs, respectively (Table S10 and S11). Pyruvate metabolism, glycolysis/gluconeogenesis, biosynthesis of phenylpropanoids were identified to be the three of the most enriched pathway and were also co-effected by both up- and down-regulated DEGs. In addition, we found taht pyruvate metabolism was shared by stage II and III, indicating the conserved and important roles of pyruvate metabolism in the dedifferentiation of immature embryo. The top ten enriched pathways in stage I, II and III samples were list in Table 3.

**Table 3:** List of first ten pathways for DEGs.

Pathway term	Pathway ID	DEGs tested	P value	Q value
**Stage I sample**				
Fatty acid biosynthesis	ko00061	25(0.42%)	0	0.0002
Metabolism of xenobiotics by cytochrome P450	ko00980	37(0.33%)	0	0.0002
alpha-Linolenic acid metabolism	ko00592	33(0.33%)	0	0.0004
Butanoate metabolism	ko00650	26(0.36%)	0	0.0005
Metabolic pathways	ko01100	570(0.18%)	0	0.001
Amino sugar and nucleotide sugar metabolism	ko00520	48(0.27%)	0.0001	0.0015
Porphyrin and chlorophyll metabolism	ko00860	20(0.37%)	0.0001	0.0019
Glutathione metabolism	ko00480	37(0.28%)	0.0002	0.0027
Linoleic acid metabolism	ko00591	21(0.33%)	0.0004	0.0054
Biosynthesis of phenylpropanoids	ko01061	153(0.20%)	0.0005	0.0062
**Stage II sample**				
Pyruvate metabolism	ko00620	47(0.39%)	0	0
Fatty acid biosynthesis	ko00061	26(0.43%)	0	0.0004
Biosynthesis of plant hormones	ko01070	164(0.26%)	0	0.0004
Glycolysis/Gluconeogenesis	ko00010	61(0.31%)	0	0.001
Biosynthesis of alkaloids derived from ornithine, lysine and nicotinic acid	ko01064	85(0.28%)	0.0001	0.0016
Amino sugar and nucleotide sugar metabolism	ko00520	55(0.31%)	0.0001	0.0016
Carbon fixation in photosynthetic organisms	ko00710	42(0.33%)	0.0001	0.0018
Metabolic pathways	ko01100	665(0.21%)	0.0003	0.0039
Biosynthesis of alkaloids derived from histidine and purine	ko01065	74(0.27%)	0.0004	0.0053
Biosynthesis of alkaloids derived from shikimate pathway	ko01063	94(0.26%)	0.0005	0.0055
**Stage III sample**				
Pyruvate metabolism	ko00620	46(0.39%)	0	0
Glycolysis/Gluconeogenesis	ko00010	63(0.32%)	0	0.0001
Phenylalanine metabolism	ko00360	61(0.31%)	0	0.0002
Biosynthesis of phenylpropanoids	ko01061	182(0.24%)	0	0.0002
Metabolic pathways	ko01100	652(0.21%)	0	0.0002
Biosynthesis of plant hormones	ko01070	159(0.25%)	0	0.0002
Biosynthesis of alkaloids derived from ornithine, lysine and nicotinic acid	ko01064	82(0.27%)	0.0001	0.001
Butanoate metabolism	ko00650	27(0.38%)	0.0001	0.001
Methane metabolism	ko00680	50(0.29%)	0.0002	0.0027
Amino sugar and nucleotide sugar metabolism	ko00520	51(0.29%)	0.0003	0.0031

### Functional analysis of DEGs

In comparison with control sample, a set of significantly expressed transcripts in stage I, stage II and stage III sample contains several genes that contribute to the dedifferentiation of maize immature. These transcripts were grouped into the category of metabolism and signal transduction based on their function annotations.

Amino acid transport and metabolism: Fourteen transcripts, related to the amino acid transport and metabolism, were all expressed in the three stages of immature embryo samples. Among them, lysine histidine transporter 1 (GRMZM2G154958), lysine histidine transporter 2 (GRMZM2G127328), nitrate transporter 1.5 (GRMZM2G061303) contribute to the transport of amino acid-related compounds. lysine histidine transporter 1 (LHT1) is revealed to play critical roles in root amino acid uptaking [20]. Lysine histidine transporter 2 has an important function in importing amino acids into the tapetum cells for synthesis of microspore-structure compounds [21]. Nitrate transporter 1.5 is important for the efficient long distance transport of nitrate [22]. Tyrosine/dopa decarboxylase (GRMZM2G093125) was responsible for catalyzing the formation of tyramine and dopamine, which is the first step in the biosynthesis of the large and diverse group of tetrahydroisoquinoline alkaloids [23]. Interestingly, it is revealed that several serine carboxypeptidase-like genes, including serine carboxypeptidase-like 50 (GRMZM2G126541), serine carboxypeptidase II-3 (GRMZM2G433767, GRMZM2G327595), and serine carboxypeptidase-like 34 (GRMZM2G161696), are identified tp be the DEGs in dedifferentiation immature samples. Several serine carboxypeptidase-like genes are belonging to the member of a family which functions as forming peptidases, acyltransferases, lyases, etc. [24], [25].

Carbohydrate transport and metabolism: There are nine transcripts identified to be associated with the carbohydrate transport and metabolism shared by stage I, stage II and stage III. Beta-glucosidase, including beta-glucosidase, chloroplastic (GRMZM2G016890, GRMZM2G008247, GRMZM2G120962), beta-glucosidase 22 (GRMZM2G055699) and beta-glucosidase 31 (GRMZM2G108133), are the most abundant transcripts in this subcategory. In addition, alpha-amylase is another enzyme associated with carbohydrate transport and metabolism.

Cell wall/membrane/envelope biogenesis: Three transcripts were associated with cell wall/membrane/envelope biogenesis functions in three samples: xylanase inhibitor protein 1 (GRMZM2G447795, GRMZM2G328171, GRMZM2G162359), acidic endochitinase (GRMZM2G130276, GRMZM2G130276) and putative lipocalin R877 (GRMZM2G072034). Galactomannan galactosyltransferase 1 is significantly differentially expressed both in stage II and III samples but not stage I. UDP-glucuronate 4-epimerase 1 (GRMZM2G455306) and UDP-glucuronate 4-epimerase 6 (GRMZM2G161233) were specific differentially significantly expressed in stage II while Xylanase inhibitor protein 2 (GRMZM2G133781) was significantly differentially expressed in stage III.

Signal transduction mechanisms: There are 22 transcripts associated with signal transduction function shared by stage I, stage II and stage III in this study. Eleven of them are kinase-related genes, including leucine-rich repeat receptor-like protein kinase (GRMZM2G459663), serine/threonine-protein kinase (GRMZM2G066432, GRMZM2G175164), wall-associated receptor kinase-like (GRMZM2G359986), CBL-interacting protein kinase (GRMZM2G390896) and PTI1-like tyrosine-protein kinase (GRMZM2G051984). Auxin-induced in root cultures protein (GRMZM2G066202 and GRMZM2G050159) is related to auxin, which plays a major role in the induction of dedifferentiation of plant tissues [26], and was identified to be significantly down-regulated in three samples except GRMZM2G050159 in stage III.

To our best knowledge, this study is the first genome-wide effort to investigate the dynamically transcriptional changes in dedifferentiation maize immature embryos. In this study, the DGE, a high-throughput Solexa/Illumina sequencing technology, was utilized to estimate gene expression and identify DEGs in libraries prepared from three stages of dedifferentiation maize immature embryos and control embryo sample. About 55% of clean tags can be mapped to ∼66% of reference genes of maize in the three stage libraries. There are two main reasons for this. Firstly, reference gene annotations in maize were still not completely finished and may contain some mis-annotations. Second, *NlaIII* site that is required for detection by DGE technology is contained by only 88% of reference genes [18], indicating that some clean tags were not identified. Although this study was a preliminary analysis of dedifferentiation of maize immature embryos, much valuable information were obtained, and those tags unmapped may represent novel genes that could be identified in the future.

High-throughput Solexa/Illumina sequencing have greatly facilitated the deferential gene expression analyses between various samples. Many genes of all the three stages of immature samples expressed highly compared to the control sample. Our data will provide valuable information for future studies of the molecular mechanisms underlying dedifferentiation of maize immature embryos and other plants.

## Supporting Information

Figure S1 **The three stage of dedifferentiation of immature embryo and control used in this study.** (A) Control. (B) Stage I. (C) Stage II and (D) Stage III.
(TIF)

Table S1 **The detailed components of N6 inducting medium.**
(DOC)

Table S2 **The primers used to perform the Real-time PCR in this study.**
(DOC)

Table S3 **List of DEGs changed for at least 5 folds in stage I sample.**
(DOC)

Table S4 **List of DEGs changed for at least 5 folds in stage II sample.**
(DOC)

Table S5 **List of DEGs changed for at least 5 folds in stage III sample.**
(DOC)

Table S6 **List of enriched pathways for up-regulated DEGs in stage I sample.**
(XLS)

Table S7 **List of enriched pathways for down-regulated DEGs in stage I sample.**
(XLS)

Table S8 **List of enriched pathways for up-regulated DEGs in stage II sample.**
(XLS)

Table S9 **List of enriched pathways for down-regulated DEGs in stage II sample.**
(XLS)

Table S10 **List of enriched pathways for up-regulated DEGs in stage III sample.**
(XLS)

Table S11 **List of enriched pathways for down-regulated DEGs in stage III sample.**
(XLS)
