# Supplementary figures and images for: Genome Expression Profile Analysis of the Immature Maize Embryo during Dedifferentiation
Source: PLoS One. 2012 Mar 20;7(3):e32237. doi: 10.1371/journal.pone.0032237 (PMC3308947; doi:10.1371/journal.pone.0032237)

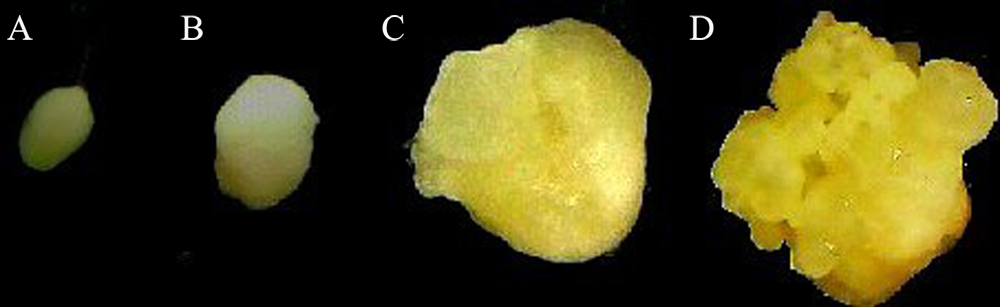

Supplement: Figure S1 — The three stage of dedifferentiation of immature embryo and control used in this study. (A) Control. (B) Stage I. (C) Stage II and (D) Stage III. (TIF) [file pone.0032237.s001.tif]
