# Supplementary material for: Genome Expression Profile Analysis of the Immature Maize Embryo during Dedifferentiation
Source: PLoS One. 2012 Mar 20;7(3):e32237. doi: 10.1371/journal.pone.0032237 (PMC3308947; doi:10.1371/journal.pone.0032237)
Supplement: Table S1 — The detailed components of N6 inducting medium. (DOC) [file pone.0032237.s002.doc]

Table S1. The detailed components of N6 inducting medium

| medium component | concentration (mg/L) |
| --- | --- |
| (NH4)_2_SO_4_ | 463.0 |
| KNO_3_ | 2830.0 |
| KH_2_PO_4_ | 400.0 |
| MgSO_4_·7H_2_O | 185.0 |
| CaCl_2_·2H_2_O | 166.0 |
| FeSO_4_·7H_2_O | 27.8 |
| Na_2_-EDTA | 37.3 |
| MnSO_4_·4H_2_O | 4.4 |
| ZnSO_4_·7H_2_O | 1.5 |
| H_3_BO_3_ | 1.6 |
| KI | 0.8 |
| Glycin | 2.0 |
| Vitamin B1 | 1.0 |
| Vitamin B6 | 0.5 |
| Nicotinic acid | 0.5 |
| Inositol | 120.0 |
| 2,4-D | 2.0 |
| Sugar | 30,000.0 |
| Powdered agar | 7,000.0 |
| PH | 5.8 |
