# Supplementary material for: Genome Expression Profile Analysis of the Immature Maize Embryo during Dedifferentiation
Source: PLoS One. 2012 Mar 20;7(3):e32237. doi: 10.1371/journal.pone.0032237 (PMC3308947; doi:10.1371/journal.pone.0032237)
Supplement: Table S2 — The primers used to perform the Real-time PCR in this study. (DOC) [file pone.0032237.s003.doc]

Table S2. The primers used to perform the Real-time PCR in this study.

| **Gene** | **Left primer** | **Right primer** |
| --- | --- | --- |
| GRMZM2G016890 | 5’-AACCCACCTATCTACATCACCG -3’ | 5’ ACCATTCAAAGTTGTCCAGCA -3’ |
| GRMZM2G071272 | 5’-AGGCTGGAAACGAACGAGG-3’ | 5’- CGACGAGCATCAATCAAATCA-3’ |
| GRMZM2G112377 | 5’- ACGCCGAACTACAACAACCG-3’ | 5’- TGTGCCTCGACAAACAACCTC-3’ |
| GRMZM2G458401 | 5’- CACCACCGACAAGCCACTAA-3’ | 5’- AAGTTTGCCCTCCACCGTCT -3’ |
| GRMZM2G474755 | 5’- CAGGCTTGTCGTCCTCGTC -3’ | 5’- CTCGGGCAGGCTGATGTAG-3’ |
| GRMZM2G086066 | 5’- GGTGAGCAGCGAGTGAGTG -3’ | 5’- CGCCTAGCAGATCCGACAG-3’ |
| GRMZM2G150276 | 5’- AGCGGCAAGGACTACTGGA-3’ | 5’- CTCCCTCCTAAGCAAGAACCA-3’ |
| GRMZM2G156877 | 5’- CGTGGATGGAGGTGGAAGC-3’ | 5’- GAAGCCGAAGTGGCAGAGG-3’ |
| GRMZM2G008247 | 5’- CTTGGAGCCTGTGGTTCGTG-3’ | 5’- CGTAGGCGTCGTCAGTGTTG-3’ |
| GRMZM2G011789 | 5’- CAGCGGCGATGCTCAAGT-3’ | 5’- GCGTAAGGCAGGTAGTGGC-3’ |
| GRMZM2G170692 | 5’- CCACCAAGTCCATTGAGCG -3’ | 5’- CAAGCCGTTGTTGTAGTAGTCG-3’ |
| GRMZM2G011789 | 5’- CAGCGGCGATGCTCAAGT -3’ | 5’- GCGTAAGGCAGGTAGTGGC-3’ |
| GRMZM2G126010 (actin1) | 5’- GAGCGGGAGATTGTCAGGG-3’ | 5’- AAGGGATGGTTGGAACAGCA-3’ |

:

:

:

:

:

（reference gene）:
