# Supplementary material for: Genome Expression Profile Analysis of the Immature Maize Embryo during Dedifferentiation
Source: PLoS One. 2012 Mar 20;7(3):e32237. doi: 10.1371/journal.pone.0032237 (PMC3308947; doi:10.1371/journal.pone.0032237)
Supplement: Table S3 — List of DEGs changed for at least 5 folds in stage I sample. (DOC) [file pone.0032237.s004.doc]

Table S3. List of DEGs changed for at least 5 folds in stage I sample.

| **Function classifications** | **Gene** | **Accession** | **Function annotations** | **Fold** |
| --- | --- | --- | --- | --- |
| **Un-regulated genes** | | | | |
| Cell wall/membrane/envelope biogenesis | GRMZM2G447795 | NP_001151661 | Xylanase inhibitor protein 1 | -14.16 |
|  | GRMZM2G130276 |  | Acidic endochitinase | -11.68 |
|  | GRMZM2G328171 | NP_001146870 | Xylanase inhibitor protein 1 | -11.3 |
|  | GRMZM2G162359 | NP_001142312 | Xylanase inhibitor protein 1 | -9.63 |
|  | GRMZM2G072034 | NP_001140887 | Putative lipocalin R877 | -9.39 |
|  | GRMZM2G453805 | NP_001105541 | Acidic endochitinase | -6.25 |
| Cytoskeleton | GRMZM2G093391 |  | Kinesin-4 | -8.21 |
| Defense mechanisms | GRMZM2G099420 |  | Dihydroflavonol-4-reductase | -11.01 |
|  | GRMZM2G013726 | NP_001140905 | Dihydroflavonol-4-reductase | -9.26 |
|  | GRMZM2G301934 | NP_001150053 | Probable carboxylesterase 18 | -8.39 |
|  | GRMZM2G131836 | NP_001105715 | Dihydroflavonol-4-reductase | -8.39 |
|  | GRMZM2G131243 | NP_001130277 | Anthocyanidin reductase | -8.39 |
| Intracellular trafficking, secretion, and vesicular transport | GRMZM2G154735 | NP_001147407 | Protein HVA22 | -10.85 |
|  | GRMZM2G023436 | NP_001131720 | Ras-related protein RABA1f | -9.39 |
| Posttranslational modification, protein turnover, chaperones | GRMZM2G150276 | NP_001105571 | Oryzain alpha chain | -11.53 |
|  | GRMZM2G156877 | NP_001105720 | Glutathione S-transferase 4 | -11.32 |
|  | GRMZM2G042639 | P49248 | Protein IN2-1 | -11.31 |
|  | GRMZM2G028129 | NP_001130836 | Probable mitochondrial chaperone BCS1-B | -10.9 |
|  | GRMZM2G021796 | NP_001151934 | RING-H2 finger protein ATL2 | -10.88 |
|  | GRMZM2G324956 | NM_001157527 | 18.9 kDa heat shock protein | -10.79 |
|  | GRMZM2G161827 |  | Probable glutathione S-transferase GSTU6 | -10.76 |
|  | GRMZM2G436084 | NP_001158966 | Thioredoxin H-type 2 | -10.11 |
|  | GRMZM2G120587 | NM_001153228 | Serine carboxypeptidase-like 51 | -9.91 |
|  | GRMZM2G429396 | NP_001147410 | 18.8 kDa class V heat shock protein | -9.76 |
|  | GRMZM2G120079 | NP_001159301 | Metacaspase-1 | -9.73 |
|  | GRMZM2G096247 | NP_001148830 | Glutathione S-transferase 6, chloroplastic | -9.69 |
|  | GRMZM2G480106 | NP_001144642 | RING-H2 finger protein ATL32 | -9.69 |
|  | GRMZM2G468657 | NP_001150678 | Aspartic proteinase nepenthesin-1 | -9.58 |
|  | GRMZM2G053206 | NP_001140805 | Basic 7S globulin | -9.51 |
|  | GRMZM2G044773 |  | E3 ubiquitin-protein ligase EL5 | -9.39 |
|  | GRMZM2G022799 | NM_001152659 | Metacaspase-9 | -9.21 |
|  | GRMZM2G340416 |  | Wall-associated receptor kinase 3 | -9.16 |
|  | GRMZM2G105523 | NP_001143732 | Pyrrolidone-carboxylate peptidase | -8.95 |
|  | GRMZM2G175593 | NP_001147824 | Probable mitochondrial chaperone bcs1 | -8.95 |
|  | GRMZM2G010491 | NP_001167771 | Thioredoxin H2-1 | -8.89 |
|  | GRMZM2G364127 |  | RING-H2 finger protein ATL5 | -8.82 |
|  | GRMZM2G448368 |  | Chaperone protein dnaJ 11, chloroplastic | -8.76 |
|  | GRMZM2G338131 | NP_001147616 | Protein IN2-1 homolog B | -8.69 |
|  | GRMZM2G089506 | NP_001168410 | Aspartic proteinase nepenthesin-2 | -8.62 |
|  | GRMZM2G367701 | NP_001150119 | Xylem cysteine proteinase 2 | -8.62 |
|  | GRMZM2G322819 |  | Prolyl endopeptidase | -8.62 |
|  | GRMZM2G025190 | B6T033 | Probable glutathione S-transferase GSTU6 | -8.61 |
|  | GRMZM2G300589 | NP_001144032 | E3 ubiquitin-protein ligase RING1-like | -8.55 |
|  | GRMZM2G144197 | NP_001150338 | Probable mitochondrial chaperone bcs1 | -8.47 |
|  | GRMZM2G118366 | NP_001145886 | Uncharacterized protein At5g39865 | -8.3 |
|  | GRMZM2G014055 | NP_001147412 | Thioredoxin H-type | -8.3 |
|  | GRMZM2G097856 |  | 14-3-3-like protein GF14-D | -8.11 |
|  | GRMZM2G066326 | NP_001149806 | Xylem cysteine proteinase 2 | -7.49 |
|  | GRMZM2G434541 |  | Probable glutathione S-transferase GSTU6 | -6.34 |
|  | GRMZM2G073465 | NP_001149658 | Oryzain alpha chain | -5.51 |
|  | GRMZM2G428168 | NP_001104987 | Probable glutathione S-transferase | -5.42 |
|  | GRMZM2G028556 | NP_001105593 | Probable glutathione S-transferase | -5.24 |
|  | GRMZM2G040515 | NM_001154835 | Pyrrolidone-carboxylate peptidase | -5.23 |
|  | GRMZM2G428179 | NP_001148308 | RING-H2 finger protein ATL40 | -5.23 |
| Signal transduction mechanisms | GRMZM2G138355 | NP_001151118 | Nudix hydrolase 13, mitochondrial | -10.28 |
|  | GRMZM2G459663 | NP_001152636 | Probable calcium-binding protein CML45 | -10.13 |
|  | GRMZM2G043799 | NP_001151810 | Probable leucine-rich repeat receptor-like protein kinase At4g00330 | -10.03 |
|  | GRMZM2G066432 | NP_001148201 | Serine/threonine-protein kinase At3g07070 | -9.58 |
|  | GRMZM2G401664 | B4FUX3 | 1-phosphatidylinositol phosphodiesterase | -9.58 |
|  | GRMZM2G025579 |  | Histidine kinase 5 | -9.51 |
|  | GRMZM2G359986 | NP_001168336 | Wall-associated receptor kinase-like 20 | -9.51 |
|  | GRMZM2G028568 | NP_001170444 | Probable serine/threonine-protein kinase At1g18390 | -9.26 |
|  | GRMZM2G050959 |  | Disease resistance protein RPM1 | -9.16 |
|  | GRMZM2G390896 | NP_001105966 | CBL-interacting protein kinase 5 | -9 |
|  | GRMZM2G159908 | NP_001131825 | Putative serine/threonine-protein kinase-like protein CCR3 | -8.69 |
|  | GRMZM2G175164 |  | Serine/threonine-protein kinase At5g01020 | -8.62 |
|  | GRMZM2G302279 |  | Putative disease resistance protein RGA1 | -8.55 |
|  | GRMZM2G026189 |  | Putative disease resistance protein RGA3 | -8.47 |
|  | GRMZM2G071759 |  | Oligopeptide transporter 7 | -8.47 |
|  | GRMZM2G062673 | NP_001147205 | Probable calcium-binding protein CML22 | -8.3 |
|  | GRMZM2G066202 | NP_001152288 | Auxin-induced in root cultures protein 12 | -8 |
|  | GRMZM2G114093 | NP_001147870 | Serine/threonine-protein kinase HT1 | -8 |
|  | GRMZM2G051984 | NP_001141331 | PTI1-like tyrosine-protein kinase 3 | -8 |
|  | GRMZM2G038893 | NP_001147438 | Wall-associated receptor kinase-like 14 | -8 |
|  | GRMZM2G050159 | NP_001148205 | Auxin-induced in root cultures protein 12 | -5.93 |
|  | GRMZM2G381071 | NP_001142366 | Polcalcin Phl p 7 | -5.32 |
| RNA processing and modification | GRMZM2G141322 | NP_001151299 | Ribonuclease 1 | -10.64 |
| Transcription | GRMZM2G139535 | NP_001150318 | Heat stress transcription factor B-1 | -11.5 |
|  | GRMZM2G095598 |  | Zinc finger protein CONSTANS-LIKE 3 | -10.18 |
|  | GRMZM2G050305 | NP_001105949 | Myb-related protein Hv1 | -9.73 |
|  | GRMZM2G134260 |  | Homeobox-leucine zipper protein HOX2 | -9.58 |
|  | GRMZM2G041462 | C4J8W1 | Homeobox-leucine zipper protein HOX6 | -9.21 |
|  | GRMZM2G047600 |  | Myb-related protein 305 | -8.82 |
|  | GRMZM2G176327 | NP_001131296 | Transcription factor MYB21 | -8.69 |
|  | GRMZM2G156348 | NP_001149390 | Trihelix transcription factor GT-3b | -8.39 |
|  | GRMZM2G106558 | NP_001130436 | Protein ODORANT1 | -8.39 |
|  | GRMZM2G004641 | NP_001146740 | BEL1-like homeodomain protein 7 | -8.39 |
|  | GRMZM2G117164 | NP_001151825 | Homeobox-leucine zipper protein HOX24 | -8.39 |
|  | GRMZM2G159431 | P56659 | Homeobox protein knotted-1-like 3 | -8.21 |
|  | GRMZM2G133512 |  | DNA-directed RNA polymerase D subunit 2a | -8.21 |
|  | GRMZM2G097349 | NP_001151416 | Homeobox-leucine zipper protein HOX23 | -8.11 |
|  | GRMZM2G139073 | NP_001104951 | MADS-box transcription factor 16 | -7.57 |
|  | GRMZM2G002131 |  | Heat stress transcription factor B-1 | -6.18 |
|  | GRMZM2G148772 |  | Zinc finger protein CONSTANS-LIKE 3 | -5.39 |
|  | GRMZM2G139284 | NP_001150967 | Myb-related protein 306 | -5.23 |
| Translation, ribosomal structure and biogenesis | GRMZM2G078396 | NP_001148342 | 60S ribosomal protein L23 | -8.47 |
| Amino acid transport and metabolism | GRMZM2G048434 | NP_001136826 | Uncharacterized membrane protein At1g06890 | -11.52 |
|  | GRMZM2G093125 | NP_001152297 | Tyrosine/DOPA decarboxylase 2 | -10.82 |
|  | GRMZM2G116554 | NP_001146361 | Uncharacterized amino-acid permease C15C4.04c | -10.45 |
|  | GRMZM2G009400 | NP_001147982 | Tyrosine/DOPA decarboxylase 2 | -10.11 |
|  | GRMZM2G127328 | NP_001147827 | Lysine histidine transporter 2 | -10.06 |
|  | GRMZM2G085381 | P42390 | Indole-3-glycerol phosphate lyase, chloroplastic | -9.62 |
|  | GRMZM2G125923 | NP_001146088 | Arogenate dehydratase/prephenate dehydratase 6, chloroplastic | -9.58 |
|  | GRMZM2G061303 |  | Nitrate transporter 1.5 | -9.51 |
|  | GRMZM2G112039 |  | Probable peptide/nitrate transporter At1g59740 | -9.43 |
|  | GRMZM2G126541 |  | Serine carboxypeptidase-like 50 | -8.89 |
|  | GRMZM2G042933 | NP_001137042 | Amino acid permease 2 | -8.82 |
|  | GRMZM2G154958 |  | Lysine histidine transporter 1 | -8.62 |
|  | GRMZM2G433767 | NP_001147904 | Serine carboxypeptidase II-3 | -8.55 |
|  | GRMZM2G111164 | NP_001132769 | Probable bifunctional methylthioribulose-1-phosphate dehydratase/enolase-phosphatase E1 | -8.47 |
|  | GRMZM2G046601 | NM_001111827 | Glutamine synthetase root isozyme 5 | -8.47 |
|  | GRMZM2G322314 | NP_001148578 | Cysteine desulfurase 1, mitochondrial | -8.3 |
|  | GRMZM2G161696 | NP_001152245 | Serine carboxypeptidase-like 34 | -8.21 |
|  | GRMZM2G036708 | NP_001136599 | Cysteine synthase, chloroplastic/chromoplastic | -7.85 |
|  | GRMZM2G327595 | NP_001147829 | Serine carboxypeptidase II-3 | -6.05 |
|  | GRMZM2G092945 | NP_001169382 | Amino acid permease 5 | -5.22 |
| Carbohydrate transport and metabolism | GRMZM2G016890 | P49235 | Beta-glucosidase, chloroplastic | -15.43 |
|  | GRMZM2G008247 | NP_001105892 | Beta-glucosidase, chloroplastic | -11.25 |
|  | GRMZM2G138468 | NP_001105539 | Alpha-amylase isozyme 3B | -10.71 |
|  | GRMZM2G055699 | NP_001145839 | Beta-glucosidase 22 | -10.45 |
|  | GRMZM2G081843 | Q9AR14 | Aquaporin PIP1-5 | -9.94 |
|  | GRMZM2G108133 | C4JAJ7 | Beta-glucosidase 31 | -9.43 |
|  | GRMZM2G031581 |  | Glycogenin-2 | -9.26 |
|  | GRMZM2G007263 |  | Glyceraldehyde-3-phosphate dehydrogenase B, chloroplastic | -8.82 |
|  | GRMZM2G394450 | P49175 | Beta-fructofuranosidase 1 | -8.55 |
|  | GRMZM2G115124 | NP_001149057 | GDP-mannose 4,6 dehydratase 2 | -8.3 |
|  | GRMZM2G120962 |  | Beta-glucosidase, chloroplastic | -8.11 |
|  | GRMZM2G031169 | NP_001151520 | Uncharacterized protein At2g34460, chloroplastic | -8 |
|  | GRMZM2G103055 | NP_001150278 | Alpha-amylase | -5.48 |
|  | GRMZM2G089836 | B6T0A9 | Acid beta-fructofuranosidase | -5.33 |
|  | GRMZM2G041356 | NP_001137118 | Aldose 1-epimerase | -5.32 |
|  | GRMZM2G154628 | Q9ATM6 | Aquaporin PIP2-4 | -5.06 |
| Energy production and conversion | GRMZM2G179063 | NP_001168657 | Flavonol-3-O-glycoside-7-O-glucosyltransferase 1 | -13.47 |
|  | GRMZM2G118800 | NP_001168661 | Aldehyde dehydrogenase family 3 member H1 | -13.35 |
|  | GRMZM2G009045 |  | Phosphate carrier protein, mitochondrial | -13.22 |
|  | GRMZM2G168474 | Q93XP7 | Cis-zeatin O-glucosyltransferase 1 | -11.96 |
|  | GRMZM2G078465 | NP_001150551 | Indole-3-acetate beta-glucosyltransferase | -11.86 |
|  | GRMZM2G159724 | NP_001152396 | NADP-dependent malic enzyme | -11.69 |
|  | GRMZM2G041699 | NP_001149205 | Cytokinin-O-glucosyltransferase 2 | -10.49 |
|  | GRMZM2G167220 | NP_001105163 | Cytokinin dehydrogenase 4 | -10.11 |
|  | GRMZM2G130119 |  | Anthocyanidin 5,3-O-glucosyltransferase | -10.11 |
|  | GRMZM2G399338 |  | Transcription factor GTE8 | -9.76 |
|  | GRMZM2G049798 | NP_001168355 | Cytokinin-O-glucosyltransferase 2 | -9.47 |
|  | GRMZM2G061321 | NP_001150609 | Anthocyanidin 3-O-glucosyltransferase | -8.62 |
|  | GRMZM2G316030 |  | Indole-3-acetate beta-glucosyltransferase 1 | -8.55 |
|  | GRMZM2G144081 | NM_001112419 | Protein brittle-1, chloroplastic/amyloplastic | -8.21 |
|  | GRMZM2G041418 | NM_001152818 | Probable NADH dehydrogenase | -8 |
|  | GRMZM2G135385 | B6SM43 | Cytochrome b5 isoform 1 | -6 |
|  | GRMZM2G010987 | NP_001149462 | Anthocyanidin 5,3-O-glucosyltransferase | -5.4 |
|  | GRMZM2G479038 | NP_001148090 | Cytokinin-O-glucosyltransferase 3 | -5.15 |
| Inorganic ion transport and metabolism | GRMZM2G102959 | NM_001177189 | Ferredoxin--nitrite reductase, chloroplastic (Fragment) | -11.95 |
|  | GRMZM2G086066 | NP_001152036 | Superoxide dismutase 1 copper chaperone | -11.18 |
|  | GRMZM2G175140 | NP_001140828 | Ammonium transporter 1 member 1 | -10.45 |
|  | GRMZM2G057616 | C9DQ40 | Chloride channel protein CLC-a | -10.21 |
|  | GRMZM2G344163 | NP_001169702 | Putative chloride channel-like protein CLC-g | -10.16 |
|  | GRMZM2G047762 |  | Zinc transporter 5 | -10.08 |
|  | GRMZM2G176430 | NP_001169517 | Tonoplast dicarboxylate transporter | -9.06 |
|  | GRMZM2G090568 | P12365 | Catalase isozyme 2 | -9.06 |
|  | GRMZM2G080178 | NP_001132356 | Sulfate transporter 1.3 | -8.47 |
|  | GRMZM2G151406 |  | Copper-transporting ATPase RAN1 | -8.47 |
|  | GRMZM2G028736 | NP_001130456 | Ammonium transporter 1 member 2 | -8.3 |
|  | GRMZM2G093276 | NP_001148241 | Zinc transporter 8 | -5.89 |
|  | GRMZM2G042412 | NP_001152680 | Copper transporter 1 | -5.27 |
|  | GRMZM2G079348 | P18123 | Catalase isozyme 3 | -5.05 |
| Lipid transport and metabolism | GRMZM2G154523 | NP_001151190 | Patatin group A-3 | -13.45 |
|  | GRMZM2G179147 | NP_001169802 | Abscisic acid 8'-hydroxylase 1 | -12.31 |
|  | GRMZM2G048522 | NP_001130893 | Probable 4-coumarate--CoA ligase 5 | -11.23 |
|  | GRMZM2G164074 | NP_001168221 | Cytochrome P450 94A1 | -10.51 |
|  | GRMZM2G150907 | NP_001140247 | Secologanin synthase | -9.94 |
|  | GRMZM2G061969 | NP_001146559 | Phospholipase D alpha 1 | -9.79 |
|  | GRMZM2G370745 | NP_001141098 | Secologanin synthase | -9.11 |
|  | GRMZM2G172098 | NP_001136742 | Monoglyceride lipase | -8.69 |
|  | GRMZM2G349749 | NP_001140826 | Patatin group A-3 | -8.62 |
|  | GRMZM2G002142 |  | Abscisic acid 8'-hydroxylase 3 | -8.47 |
|  | GRMZM2G032896 | B6T0I8 | Cytochrome P450 90D2 | -8.3 |
|  | GRMZM2G106468 | C4J528 | Secologanin synthase | -8.3 |
|  | GRMZM2G110616 | NP_001169640 | Putative acyl-CoA synthetase YngI | -8.21 |
|  | GRMZM2G159179 |  | Cytochrome P450 94A1 | -5.3 |
| Secondary metabolites biosynthesis, transport and catabolism | GRMZM2G170692 | NP_001168086 | Phenylalanine ammonia-lyase | -12.79 |
|  | GRMZM2G170017 | NP_001147467 | Salutaridine reductase | -12.47 |
|  | GRMZM2G154870 |  | Isoflavone 2'-hydroxylase | -11.86 |
|  | GRMZM2G152975 | NP_001169684 | Alcohol dehydrogenase-like 4 | -11.78 |
|  | GRMZM2G154828 | NP_001130688 | Isoflavone 2'-hydroxylase | -11.16 |
|  | GRMZM2G354909 | NP_001146814 | (+)-neomenthol dehydrogenase | -11.09 |
|  | GRMZM2G087875 | NP_001146006 | Isoflavone 2'-hydroxylase | -11.08 |
|  | GRMZM2G085661 | Q43257 | Cytochrome P450 71C4 | -9.79 |
|  | GRMZM2G312069 | NP_001142304 | Isoflavone 2'-hydroxylase | -9.73 |
|  | GRMZM2G432480 | NP_001152673 | Protein STAR1 | -9.69 |
|  | GRMZM2G113844 | B4FUC5 | Probable flavin-containing monooxygenase 1 | -9.43 |
|  | GRMZM2G063917 | NP_001147922 | Phenylalanine ammonia-lyase | -9.16 |
|  | GRMZM2G148052 |  | Cytochrome P450 71D7 | -9.16 |
|  | GRMZM2G033952 | NP_001152511 | Probable caffeoyl-CoA O-methyltransferase At4g34050 | -8.95 |
|  | GRMZM2G172826 |  | Cytochrome P450 71A9 | -8.69 |
|  | GRMZM2G167613 | NP_001136687 | Probable cinnamyl alcohol dehydrogenase 6 | -8.69 |
|  | GRMZM2G086730 |  | ABC transporter B family member 11 | -8.55 |
|  | GRMZM2G302074 |  | Cytochrome P450 89A2 | -8.39 |
|  | GRMZM2G028677 | NP_001151365 | Trans-cinnamate 4-monooxygenase | -6.09 |
|  | GRMZM2G042660 | NM_001152492 | Primary amine oxidase | -5.26 |
| **Down-regulated genes** | | | | |
| Cell cycle control, cell division, chromosome partitioning | GRMZM2G056303 | NP_001169350 | Cyclin-D5-1 | 7.95 |
| Cell wall/membrane/envelope biogenesis | GRMZM2G008507 |  | Sucrose-phosphate synthase 2 | 5.43 |
| Cytoskeleton | GRMZM2G417410 |  | Formin-like protein 12 | 8.69 |
|  | GRMZM2G471108 |  | Myosin-J heavy chain | 8.37 |
| Defense mechanisms | GRMZM2G112792 |  | L-gulonolactone oxidase | 8.46 |
| Extracellular structures | GRMZM2G035377 | NP_001167834 | Probable glucuronosyltransferase Os03g0107900 | 8.95 |
| Intracellular trafficking, secretion, and vesicular transport | GRMZM2G473162 | NP_001151036 | Putative clathrin assembly protein At2g01600 | 8.27 |
| Posttranslational modification, protein turnover, chaperones | GRMZM2G071272 |  | Probable mitochondrial chaperone bcs1 | 10.69 |
|  | GRMZM2G346839 | NP_001149613 | 23.2 kDa heat shock protein | 9.27 |
|  | GRMZM2G136522 | NP_001136660 | Thioredoxin reductase NTRC | 8.17 |
|  | GRMZM2G084440 | NP_001150143 | Rab GDP dissociation inhibitor alpha | 8.07 |
| Signal transduction mechanisms | GRMZM2G166658 |  | CBL-interacting protein kinase 2 | 8.69 |
|  | GRMZM2G148807 |  | Glutamate receptor 3.4 | 8.37 |
|  | GRMZM2G311220 | NP_001149916 | Calcium-dependent protein kinase 13 | 8.37 |
|  | GRMZM2G332660 |  | Calcium-dependent protein kinase 13 | 7.95 |
| Replication, recombination and repair | GRMZM2G098714 | NP_001147118 | Replication protein A 70 kDa DNA-binding subunit | 7.95 |
| RNA processing and modification | GRMZM2G458401 |  | 5'-3' exoribonuclease 2 | 10.71 |
|  | GRMZM2G340618 |  | Helicase SEN1 | 7.95 |
| Transcription | GRMZM2G124663 |  | Nuclear transcription factor Y subunit B-6 | 12.47 |
|  | GRMZM2G011789 | NP_001105518 | Nuclear transcription factor Y subunit B-6 | 11.54 |
|  | GRMZM2G051528 | NP_001105937 | Transcription factor MYB12 | 9.76 |
|  | GRMZM2G088783 | NM_001154866 | Transcription factor MYB86 | 9.17 |
|  | GRMZM2G314660 |  | Trihelix transcription factor GT-2 | 8.17 |
|  | GRMZM2G163335 |  | Agamous-like MADS-box protein AGL61 | 7.95 |
|  | GRMZM2G076272 | NM_001148413 | Homeobox protein ATH1 | 7.95 |
|  | GRMZM2G112247 | NM_001155772 | Protein GDAP2 homolog | 7.26 |
| Amino acid transport and metabolism | GRMZM2G106950 | NP_001168869 | Indole-3-glycerol phosphate synthase, chloroplastic | 8.54 |
|  | GRMZM2G123815 | NP_001148579 | Serine carboxypeptidase-like 33 | 5.35 |
| Carbohydrate transport and metabolism | GRMZM2G013255 | NP_001151568 | Alpha-L-fucosidase 1 | 10.47 |
|  | GRMZM2G063048 |  | Uncharacterized protein At2g34460, chloroplastic | 8.27 |
| Coenzyme transport and metabolism | GRMZM2G161673 | NP_001141335 | Magnesium-protoporphyrin O-methyltransferase | 9.07 |
| Energy production and conversion | GRMZM2G432291 |  | Cyanohydrin beta-glucosyltransferase | 10.69 |
|  | GRMZM2G097030 | NP_001170472 | Cytokinin-N-glucosyltransferase 1 | 10.65 |
|  | GRMZM2G428027 |  | Nitrate reductase [NAD(P)H] | 10.09 |
| Inorganic ion transport and metabolism | GRMZM2G112377 | NP_001105817 | Inorganic phosphate transporter 1-6 | 14.19 |
|  | GRMZM2G370780 |  | SPX domain-containing protein 5 | 9.22 |
|  | GRMZM2G410990 |  | ATPase 6, plasma membrane-type | 8.46 |
|  | GRMZM2G037343 |  | Cation/H(+) antiporter 15 | 8.27 |
|  | GRMZM2G065989 |  | SPX domain-containing protein 6 | 7.29 |
|  | GRMZM2G171423 | NP_001136588 | SPX domain-containing protein 1 | 5.85 |
| Lipid transport and metabolism | GRMZM2G351023 |  | 3-oxoacyl-[acyl-carrier-protein] reductase 3, chloroplastic | 10.07 |
|  | GRMZM2G117064 |  | Long chain acyl-CoA synthetase 9, chloroplastic | 9.22 |
|  | GRMZM2G095757 |  | Phosphatidylserine synthase 2 | 8.76 |
|  | GRMZM2G154652 |  | Cytochrome P450 716B1 | 8.54 |
|  | GRMZM2G477503 |  | GDP-mannose-dependent alpha-mannosyltransferase | 5.64 |
| Nucleotide transport and metabolism | GRMZM2G080387 |  | Nucleobase-ascorbate transporter 2 | 9.45 |
| Secondary metabolites biosynthesis, transport and catabolism | GRMZM2G147966 |  | Respiratory burst oxidase homolog protein E | 9.01 |
|  | GRMZM2G091715 | B4FFE7 | Acyl carrier protein 3, chloroplastic | 9.01 |
|  | GRMZM2G177314 | NP_001147125 | ABC transporter G family member 11 | 8.69 |
|  | GRMZM2G091819 | NP_001105991 | Putative flavin-containing monooxygenase YUCCA11 | 8.37 |
|  | GRMZM2G359298 | NM_001175055 | Primary amine oxidase | 8.17 |
|  | GRMZM2G025222 |  | Flavin-containing monooxygenase YUCCA1 | 5.67 |
