# Supplementary material for: Genome Expression Profile Analysis of the Immature Maize Embryo during Dedifferentiation
Source: PLoS One. 2012 Mar 20;7(3):e32237. doi: 10.1371/journal.pone.0032237 (PMC3308947; doi:10.1371/journal.pone.0032237)
Supplement: Table S4 — List of DEGs changed for at least 5 folds in stage II sample. (DOC) [file pone.0032237.s005.doc]

Table S4. List of DEGs changed for at least 5 folds in stage II sample.

| **Function classification** | **Gene** | **Accession** | **Function annotation** | **Fold** |
| --- | --- | --- | --- | --- |
| **Up-regulated genes** | | | | |
| Cell cycle control, cell division, chromosome partitioning | GRMZM2G096228 | NP_001149887 | Probable calcium-binding protein CML8 | -9.43 |
|  | GRMZM2G458867 |  | Putative pleiotropic drug resistance protein 7 | -8.15 |
|  | GRMZM2G704285 |  | MORC family CW-type zinc finger protein 3 | -7.92 |
| Cell wall/membrane/envelope biogenesis | GRMZM2G447795 | NP_001151661 | Xylanase inhibitor protein 1 | -16.21 |
|  | GRMZM2G072034 | NP_001140887 | Putative lipocalin R877 | -11.71 |
|  | GRMZM2G328171 | NP_001146870 | Xylanase inhibitor protein 1 | -11.38 |
|  | GRMZM2G162359 | NP_001142312 | Xylanase inhibitor protein 1 | -10.1 |
|  | GRMZM2G161233 |  | UDP-glucuronate 4-epimerase 6 | -9.87 |
|  | GRMZM2G130276 |  | Acidic endochitinase | -9.39 |
|  | GRMZM2G435120 |  | Galactomannan galactosyltransferase 1 | -9.04 |
|  | GRMZM2G455306 |  | UDP-glucuronate 4-epimerase 1 | -7.92 |
| Cytoskeleton | GRMZM2G151934 |  | Protein DA1-related 2 | -10.49 |
|  | GRMZM2G175761 | NP_001148027 | Pollen-specific protein SF3 | -10.27 |
|  | GRMZM2G132958 | NP_001144650 | IST1-like protein | -9.1 |
|  | GRMZM2G429928 |  | Formin-like protein 4 | -8.8 |
| Defense mechanisms | GRMZM2G099420 |  | Dihydroflavonol-4-reductase | -16.68 |
|  | GRMZM2G301934 | NP_001150053 | Probable carboxylesterase 18 | -9.25 |
|  | GRMZM2G004683 | NP_001105644 | Dihydroflavonol-4-reductase | -7.92 |
| Intracellular trafficking, secretion, and vesicular transport | GRMZM2G154735 | NP_001147407 | Protein HVA22 | -10.59 |
|  | GRMZM2G023436 | NP_001131720 | Ras-related protein RABA1f | -10.12 |
|  | GRMZM2G081105 | NP_001146151 | GDP-mannose transporter GONST2 | -8.15 |
|  | GRMZM2G003769 | NP_001146842 | Peroxisomal membrane protein 11-4 | -7.92 |
|  | GRMZM2G061950 | NP_001105475 | Annexin D1 | -5.42 |
| Posttranslational modification, protein turnover, chaperones | GRMZM2G150276 | NP_001105571 | Oryzain alpha chain | -14.51 |
|  | GRMZM2G120079 | NP_001159301 | Metacaspase-1 | -12.65 |
|  | GRMZM2G480106 | NP_001144642 | RING-H2 finger protein ATL32 | -12.64 |
|  | GRMZM2G324956 | NM_001157527 | 18.9 kDa heat shock protein | -12.57 |
|  | GRMZM2G118366 | NP_001145886 | Uncharacterized protein At5g39865 | -12.4 |
|  | GRMZM2G448368 |  | Chaperone protein dnaJ 11, chloroplastic | -11.41 |
|  | GRMZM2G124684 | NP_001150335 | Aspartic proteinase nepenthesin-2 | -11.24 |
|  | GRMZM2G010491 | NP_001167771 | Thioredoxin H2-1 | -11.19 |
|  | GRMZM2G429396 | NP_001147410 | 18.8 kDa class V heat shock protein | -10.57 |
|  | GRMZM2G028129 | NP_001130836 | Probable mitochondrial chaperone BCS1-B | -10.47 |
|  | GRMZM2G014055 | NP_001147412 | Thioredoxin H-type | -10.17 |
|  | GRMZM2G156877 | NP_001105720 | Glutathione S-transferase 4 | -10.07 |
|  | GRMZM2G105523 | NP_001143732 | Pyrrolidone-carboxylate peptidase | -9.96 |
|  | GRMZM2G096247 | NP_001148830 | Glutathione S-transferase 6, chloroplastic | -9.93 |
|  | GRMZM2G120587 | NM_001153228 | Serine carboxypeptidase-like 51 | -9.63 |
|  | GRMZM2G340416 |  | Wall-associated receptor kinase 3 | -9.51 |
|  | GRMZM2G022799 | NM_001152659 | Metacaspase-9 | -9.43 |
|  | GRMZM2G300589 | NP_001144032 | E3 ubiquitin-protein ligase RING1-like | -9.34 |
|  | GRMZM2G021796 | NP_001151934 | RING-H2 finger protein ATL2 | -9.2 |
|  | GRMZM2G401848 | NP_001149128 | Peptidyl-prolyl cis-trans isomerase | -9.15 |
|  | GRMZM2G053206 | NP_001140805 | Basic 7S globulin | -9.07 |
|  | GRMZM2G436084 | NP_001158966 | Thioredoxin H-type 2 | -8.86 |
|  | GRMZM2G106165 | NP_001148149 | Serine carboxypeptidase-like 51 | -8.8 |
|  | GRMZM2G152587 |  | Probable mitochondrial chaperone BCS1-B | -8.43 |
|  | GRMZM2G044773 |  | E3 ubiquitin-protein ligase EL5 | -8.34 |
|  | GRMZM2G121111 |  | Myb-like protein J | -8.25 |
|  | GRMZM2G468657 | NP_001150678 | Aspartic proteinase nepenthesin-1 | -8.25 |
|  | GRMZM2G161827 |  | Probable glutathione S-transferase GSTU6 | -8.04 |
|  | GRMZM2G175593 | NP_001147824 | Probable mitochondrial chaperone bcs1 | -8.04 |
|  | GRMZM2G129146 | NP_001130675 | Aspartic proteinase nepenthesin-2 | -7.92 |
|  | GRMZM2G098102 | NP_001130503 | Cysteine proteinase EP-B 1 | -7.92 |
|  | GRMZM2G428179 | NP_001148308 | RING-H2 finger protein ATL40 | -7.6 |
|  | GRMZM2G025190 | B6T033 | Probable glutathione S-transferase GSTU6 | -6.97 |
|  | GRMZM2G040803 | NP_001152715 | E3 ubiquitin-protein ligase RMA1H1 | -6.79 |
|  | GRMZM2G040515 | NM_001154835 | Pyrrolidone-carboxylate peptidase | -6.4 |
|  | GRMZM2G073465 | NP_001149658 | Oryzain alpha chain | -6.17 |
|  | GRMZM2G066326 | NP_001149806 | Xylem cysteine proteinase 2 | -5.76 |
|  | GRMZM2G323757 |  | Basic 7S globulin | -5.42 |
|  | GRMZM2G434541 |  | Probable glutathione S-transferase GSTU6 | -5.32 |
|  | GRMZM2G035045 | NP_001140873 | Cysteine proteinase EP-B 2 | -5.3 |
|  | GRMZM2G091201 | NP_001151615 | Transcription factor MYB1R1 | -5.13 |
| Signal transduction mechanisms | GRMZM2G330049 | NP_001152211 | CBL-interacting protein kinase 16 | -10.9 |
|  | GRMZM2G401664 | B4FUX3 | 1-phosphatidylinositol phosphodiesterase | -10.8 |
|  | GRMZM2G043799 | NP_001151810 | Probable leucine-rich repeat receptor-like protein kinase At4g00330 | -10.73 |
|  | GRMZM2G459663 | NP_001152636 | Probable calcium-binding protein CML45 | -10.63 |
|  | GRMZM2G050959 |  | Disease resistance protein RPM1 | -10.34 |
|  | GRMZM2G025579 |  | Histidine kinase 5 | -9.7 |
|  | GRMZM2G066432 | NP_001148201 | Serine/threonine-protein kinase At3g07070 | -9.55 |
|  | GRMZM2G141975 | NM_001165629 | Oligopeptide transporter 4 | -9.51 |
|  | GRMZM2G359986 | NP_001168336 | Wall-associated receptor kinase-like 20 | -9.47 |
|  | GRMZM2G062673 | NP_001147205 | Probable calcium-binding protein CML22 | -9.39 |
|  | GRMZM2G390896 | NP_001105966 | CBL-interacting protein kinase 5 | -9.34 |
|  | GRMZM2G159908 | NP_001131825 | Putative serine/threonine-protein kinase-like protein CCR3 | -9.3 |
|  | GRMZM2G165060 | NP_001141571 | Probable LRR receptor-like serine/threonine-protein kinase At1g56140 | -8.99 |
|  | GRMZM2G161380 | NP_001169946 | Serine/threonine-protein kinase-like protein ACR4 | -8.8 |
|  | GRMZM2G156255 |  | Potassium channel SKOR | -8.59 |
|  | GRMZM2G066202 | NP_001152288 | Auxin-induced in root cultures protein 12 | -8.59 |
|  | GRMZM2G028568 | NP_001170444 | Probable serine/threonine-protein kinase At1g18390 | -8.51 |
|  | GRMZM2G175164 |  | Serine/threonine-protein kinase At5g01020 | -8.51 |
|  | GRMZM2G048475 |  | Adenylyl cyclase-associated protein | -8.43 |
|  | GRMZM2G005557 |  | Probable calcium-binding protein CML9 | -8.15 |
|  | GRMZM2G138355 | NP_001151118 | Nudix hydrolase 13, mitochondrial | -8.15 |
|  | GRMZM2G152901 |  | Receptor-like serine/threonine-protein kinase At3g01300 | -8.15 |
|  | GRMZM2G110968 | NP_001140459 | Serine/threonine-protein kinase At5g01020 | -8.15 |
|  | GRMZM2G053833 | NP_001148333 | Probable calcium-binding protein CML45 | -8.04 |
|  | GRMZM2G147373 | NP_001141346 | Serine/threonine-protein kinase At5g01020 | -8.04 |
|  | GRMZM2G005452 | NP_001105809 | Disease resistance protein RPM1 | -7.92 |
|  | GRMZM2G381071 | NP_001142366 | Polcalcin Phl p 7 | -6.16 |
|  | GRMZM2G050159 | NP_001148205 | Auxin-induced in root cultures protein 12 | -5.81 |
|  | GRMZM2G061537 |  | Probable leucine-rich repeat receptor-like protein kinase At4g00330 | -5.74 |
|  | GRMZM2G059671 | NP_001130400 | Serine/threonine-protein kinase CTR1 | -5.36 |
|  | GRMZM2G379540 | NP_001140268 | Rhomboid family member 1 | -5.14 |
| Chromatin structure and dynamics | GRMZM2G170201 | B6UI28 | Transcriptional regulatory protein SIN3 | -8.8 |
|  | GRMZM2G081474 | NP_001143835 | Probable histone deacetylase 19 | -8.25 |
| RNA processing and modification | GRMZM2G141322 | NP_001151299 | Ribonuclease 1 | -9.34 |
|  | GRMZM2G322506 |  | Helicase SEN1 | -9.04 |
|  | GRMZM2G404375 | NM_001174609 | Ribonuclease J | -8.51 |
| Transcription | GRMZM2G041462 | C4J8W1 | Homeobox-leucine zipper protein HOX6 | -11.99 |
|  | GRMZM2G095598 |  | Zinc finger protein CONSTANS-LIKE 3 | -11.16 |
|  | GRMZM2G139535 | NP_001150318 | Heat stress transcription factor B-1 | -11.15 |
|  | GRMZM2G050305 | NP_001105949 | Myb-related protein Hv1 | -9.59 |
|  | GRMZM2G134260 |  | Homeobox-leucine zipper protein HOX2 | -8.86 |
|  | GRMZM2G137046 | NP_001152483 | Transcription factor HY5 | -8.8 |
|  | GRMZM2G139073 | NP_001104951 | MADS-box transcription factor 16 | -8.77 |
|  | GRMZM2G458728 |  | BEL1-like homeodomain protein 1 | -8.34 |
|  | GRMZM2G097349 | NP_001151416 | Homeobox-leucine zipper protein HOX23 | -8.25 |
|  | GRMZM2G034113 | NP_001149510 | Homeobox-leucine zipper protein HOX6 | -8.04 |
|  | GRMZM2G117164 | NP_001151825 | Homeobox-leucine zipper protein HOX24 | -8.04 |
|  | GRMZM2G171466 | NP_001147705 | Nascent polypeptide-associated complex subunit alpha-like protein 2 | -8.04 |
|  | GRMZM2G148772 |  | Zinc finger protein CONSTANS-LIKE 3 | -5.84 |
|  | GRMZM2G106276 | NP_001150756 | Homeobox-leucine zipper protein HOX28 | -5.8 |
|  | GRMZM2G419239 | NP_001106009 | Myb-related protein Zm38 | -5.43 |
|  | GRMZM2G011588 | NP_001147963 | BEL1-like homeodomain protein 6 | -5.03 |
| Amino acid transport and metabolism | GRMZM2G048434 | NP_001136826 | Uncharacterized membrane protein At1g06890 | -12.27 |
|  | GRMZM2G085381 | P42390 | Indole-3-glycerol phosphate lyase, chloroplastic | -12.08 |
|  | GRMZM2G042933 | NP_001137042 | Amino acid permease 2 | -11.83 |
|  | GRMZM2G178734 |  | Probable sugar phosphate/phosphate translocator At5g25400 | -10.43 |
|  | GRMZM2G112039 |  | Probable peptide/nitrate transporter At1g59740 | -10.04 |
|  | GRMZM2G433767 | NP_001147904 | Serine carboxypeptidase II-3 | -9.99 |
|  | GRMZM2G125923 | NP_001146088 | Arogenate dehydratase/prephenate dehydratase 6, chloroplastic | -9.73 |
|  | GRMZM2G126541 |  | Serine carboxypeptidase-like 50 | -9.7 |
|  | GRMZM2G009400 | NP_001147982 | Tyrosine/DOPA decarboxylase 2 | -9.7 |
|  | GRMZM2G089140 |  | Probable nitrite transporter At1g68570 | -9.25 |
|  | GRMZM2G127328 | NP_001147827 | Lysine histidine transporter 2 | -8.99 |
|  | GRMZM2G173597 | NP_001150301 | Probable amino acid permease 7 | -8.67 |
|  | GRMZM2G116554 | NP_001146361 | Uncharacterized amino-acid permease C15C4.04c | -8.59 |
|  | GRMZM2G154958 |  | Lysine histidine transporter 1 | -8.43 |
|  | GRMZM2G046601 | NM_001111827 | Glutamine synthetase root isozyme 5 | -8.43 |
|  | GRMZM2G161696 | NP_001152245 | Serine carboxypeptidase-like 34 | -8.34 |
|  | GRMZM2G061303 |  | Nitrate transporter 1.5 | -8.25 |
|  | GRMZM2G069203 | NP_001105082 | Probable serine acetyltransferase 1 | -8.15 |
|  | GRMZM2G111164 | NP_001132769 | Probable bifunctional methylthioribulose-1-phosphate dehydratase/enolase-phosphatase E1 | -8.04 |
|  | GRMZM2G036708 | NP_001136599 | Cysteine synthase, chloroplastic/chromoplastic | -7.4 |
|  | GRMZM2G327595 | NP_001147829 | Serine carboxypeptidase II-3 | -6.61 |
|  | GRMZM2G092945 | NP_001169382 | Amino acid permease 5 | -5.25 |
|  | GRMZM2G065974 |  | Uncharacterized amino acid permease YfnA | -5.19 |
|  | GRMZM2G055452 |  | Glutamate decarboxylase 1 | -5.06 |
|  | GRMZM2G098875 | NP_001167941 | Glutamate decarboxylase 1 | -5.01 |
| Carbohydrate transport and metabolism | GRMZM2G016890 | P49235 | Beta-glucosidase, chloroplastic | -14.85 |
|  | GRMZM2G008247 | NP_001105892 | Beta-glucosidase, chloroplastic | -13.6 |
|  | GRMZM2G120962 |  | Beta-glucosidase, chloroplastic | -12.9 |
|  | GRMZM2G138468 | NP_001105539 | Alpha-amylase isozyme 3B | -12.64 |
|  | GRMZM2G115124 | NP_001149057 | GDP-mannose 4,6 dehydratase 2 | -12.42 |
|  | GRMZM2G081843 | Q9AR14 | Aquaporin PIP1-5 | -12.17 |
|  | GRMZM2G007263 |  | Glyceraldehyde-3-phosphate dehydrogenase B, chloroplastic | -11.21 |
|  | GRMZM2G401970 |  | 6-phosphofructokinase 3 | -10.32 |
|  | GRMZM2G055699 | NP_001145839 | Beta-glucosidase 22 | -9.63 |
|  | GRMZM2G081192 | Q9ATM7 | Aquaporin PIP2-3 | -9.63 |
|  | GRMZM2G174807 | Q41870 | Probable aquaporin PIP1-2 | -9.15 |
|  | GRMZM2G108133 | C4JAJ7 | Beta-glucosidase 31 | -8.86 |
|  | GRMZM2G031169 | NP_001151520 | Uncharacterized protein At2g34460, chloroplastic | -8.15 |
|  | GRMZM2G048230 |  | Trehalose-phosphate phosphatase | -8.04 |
|  | GRMZM2G154628 | Q9ATM6 | Aquaporin PIP2-4 | -6.22 |
|  | GRMZM2G103055 | NP_001150278 | Alpha-amylase | -5.25 |
|  | GRMZM2G143187 |  | Adenosine 3'-phospho 5'-phosphosulfate transporter 2 | -5.2 |
| Coenzyme transport and metabolism | GRMZM2G038821 | Q05326 | Pyruvate decarboxylase isozyme 1 | -5.7 |
| Energy production and conversion | GRMZM2G168474 | Q93XP7 | Cis-zeatin O-glucosyltransferase 1 | -12.73 |
|  | GRMZM2G118800 | NP_001168661 | Aldehyde dehydrogenase family 3 member H1 | -12.59 |
|  | GRMZM2G159724 | NP_001152396 | NADP-dependent malic enzyme | -12.27 |
|  | GRMZM2G009045 |  | Phosphate carrier protein, mitochondrial | -11.7 |
|  | GRMZM2G167220 | NP_001105163 | Cytokinin dehydrogenase 4 | -11.42 |
|  | GRMZM2G041699 | NP_001149205 | Cytokinin-O-glucosyltransferase 2 | -10.9 |
|  | GRMZM2G179063 | NP_001168657 | Flavonol-3-O-glycoside-7-O-glucosyltransferase 1 | -10.63 |
|  | GRMZM2G144081 | NM_001112419 | Protein brittle-1, chloroplastic/amyloplastic | -10.12 |
|  | GRMZM2G078465 | NP_001150551 | Indole-3-acetate beta-glucosyltransferase | -9.39 |
|  | GRMZM2G130119 |  | Anthocyanidin 5,3-O-glucosyltransferase | -9.04 |
|  | GRMZM2G316030 |  | Indole-3-acetate beta-glucosyltransferase 1 | -8.8 |
|  | GRMZM2G049798 | NP_001168355 | Cytokinin-O-glucosyltransferase 2 | -8.73 |
|  | GRMZM2G399338 |  | Transcription factor GTE8 | -8.59 |
|  | GRMZM2G061321 | NP_001150609 | Anthocyanidin 3-O-glucosyltransferase | -8.15 |
|  | GRMZM2G098890 | NP_001170719 | Cytokinin-O-glucosyltransferase 2 | -7.92 |
|  | GRMZM2G142386 |  | Nitrate reductase [NADH] 1 | -6.31 |
|  | GRMZM2G173536 | C0P676 | SNF1-related protein kinase regulatory subunit gamma 1 | -6.15 |
|  | GRMZM2G010987 | NP_001149462 | Anthocyanidin 5,3-O-glucosyltransferase | -5.44 |
|  | GRMZM2G479038 | NP_001148090 | Cytokinin-O-glucosyltransferase 3 | -5.03 |
| Inorganic ion transport and metabolism | GRMZM2G086066 | NP_001152036 | Superoxide dismutase 1 copper chaperone | -13.28 |
|  | GRMZM2G102959 | NM_001177189 | Ferredoxin--nitrite reductase, chloroplastic (Fragment) | -11.78 |
|  | GRMZM2G090568 | P12365 | Catalase isozyme 2 | -11.56 |
|  | GRMZM2G344163 | NP_001169702 | Putative chloride channel-like protein CLC-g | -10.47 |
|  | GRMZM2G057616 | C9DQ40 | Chloride channel protein CLC-a | -10.04 |
|  | GRMZM2G047762 |  | Zinc transporter 5 | -9.63 |
|  | GRMZM2G175140 | NP_001140828 | Ammonium transporter 1 member 1 | -9.55 |
|  | GRMZM2G079381 | P17847 | Ferredoxin--nitrite reductase, chloroplastic (Fragment) | -9.55 |
|  | GRMZM2G151406 |  | Copper-transporting ATPase RAN1 | -8.86 |
|  | GRMZM2G175576 |  | Cadmium/zinc-transporting ATPase 3 | -8.73 |
|  | GRMZM2G046480 |  | Vacuolar cation/proton exchanger 2 | -8.59 |
|  | GRMZM2G115190 |  | Fe(2+) transport protein 2 | -8.51 |
|  | GRMZM2G080178 | NP_001132356 | Sulfate transporter 1.3 | -7.92 |
|  | GRMZM2G096365 | NP_001170245 | Chloride channel protein CLC-c | -5.35 |
| Lipid transport and metabolism | GRMZM2G179147 | NP_001169802 | Abscisic acid 8'-hydroxylase 1 | -13.04 |
|  | GRMZM2G154523 | NP_001151190 | Patatin group A-3 | -12.88 |
|  | GRMZM2G150907 | NP_001140247 | Secologanin synthase | -11.04 |
|  | GRMZM2G057136 |  | Triacylglycerol lipase 2 | -10.7 |
|  | GRMZM2G164074 | NP_001168221 | Cytochrome P450 94A1 | -10.2 |
|  | GRMZM2G032896 | B6T0I8 | Cytochrome P450 90D2 | -10.15 |
|  | GRMZM2G370745 | NP_001141098 | Secologanin synthase | -9.99 |
|  | GRMZM2G002142 |  | Abscisic acid 8'-hydroxylase 3 | -9.51 |
|  | GRMZM2G363429 | C4J505 | Cytochrome P450 26C1 | -9.04 |
|  | GRMZM2G061969 | NP_001146559 | Phospholipase D alpha 1 | -8.99 |
|  | GRMZM2G024144 |  | Lipid phosphate phosphatase 2 | -8.8 |
|  | GRMZM2G349749 | NP_001140826 | Patatin group A-3 | -8.8 |
|  | GRMZM2G126083 |  | Monoglyceride lipase | -8.67 |
|  | GRMZM2G110616 | NP_001169640 | Putative acyl-CoA synthetase YngI | -8.67 |
|  | GRMZM2G172098 | NP_001136742 | Monoglyceride lipase | -8.15 |
|  | GRMZM2G006937 | NP_001152006 | Cycloartenol synthase | -8.04 |
|  | GRMZM2G471240 |  | Patatin group A-3 | -7.92 |
|  | GRMZM2G396248 |  | Cytochrome P450 94A1 | -5.6 |
| Nucleotide transport and metabolism | GRMZM2G066923 | NP_001151494 | Equilibrative nucleoside transporter 4 | -5.1 |
| Secondary metabolites biosynthesis, transport and catabolism | GRMZM2G170017 | NP_001147467 | Salutaridine reductase | -11.71 |
|  | GRMZM2G085661 | Q43257 | Cytochrome P450 71C4 | -11.55 |
|  | GRMZM2G170692 | NP_001168086 | Phenylalanine ammonia-lyase | -11.41 |
|  | GRMZM2G152975 | NP_001169684 | Alcohol dehydrogenase-like 4 | -10.99 |
|  | GRMZM2G312069 | NP_001142304 | Isoflavone 2'-hydroxylase | -10.53 |
|  | GRMZM2G432480 | NP_001152673 | Protein STAR1 | -10.36 |
|  | GRMZM2G087875 | NP_001146006 | Isoflavone 2'-hydroxylase | -10.22 |
|  | GRMZM2G354909 | NP_001146814 | (+)-neomenthol dehydrogenase | -9.9 |
|  | GRMZM2G113844 | B4FUC5 | Probable flavin-containing monooxygenase 1 | -9.43 |
|  | GRMZM2G063917 | NP_001147922 | Phenylalanine ammonia-lyase | -8.99 |
|  | GRMZM2G148052 |  | Cytochrome P450 71D7 | -8.43 |
|  | GRMZM2G033952 | NP_001152511 | Probable caffeoyl-CoA O-methyltransferase At4g34050 | -8.34 |
|  | GRMZM2G088601 | NP_001158893 | Phytoene dehydrogenase, chloroplastic/chromoplastic | -8.34 |
|  | GRMZM2G028677 | NP_001151365 | Trans-cinnamate 4-monooxygenase | -6.64 |
|  | GRMZM2G160541 |  | Phenylalanine ammonia-lyase | -6.1 |
|  | GRMZM2G031117 |  | Laccase-7 | -5.57 |
|  | GRMZM2G074604 | NP_001105334 | Phenylalanine ammonia-lyase | -5.32 |
|  | GRMZM2G446858 | C4PJN4 | Carotenoid cleavage dioxygenase 8, chloroplastic | -5.19 |
|  | GRMZM2G300965 | NP_001167766 | Respiratory burst oxidase homolog protein B | -5.09 |
| **Down-regulated genes** | | | | |
| Cell cycle control, cell division, chromosome partitioning | GRMZM2G056303 | NP_001169350 | Cyclin-D5-1 | 7.95 |
| Cytoskeleton | GRMZM2G417410 |  | Formin-like protein 12 | 8.69 |
| Defense mechanisms | GRMZM2G112792 |  | L-gulonolactone oxidase | 8.46 |
|  | GRMZM2G026095 |  | Probable carboxylesterase 12 | 8.17 |
|  | GRMZM2G391795 | C4J9M8 | Probable carboxylesterase 15 | 5.55 |
| Posttranslational modification, protein turnover, chaperones | GRMZM2G032763 | NP_001151099 | Uncharacterized protein At5g39865 | 8.27 |
|  | GRMZM2G122003 | C4J8R4 | Probable ubiquitin-conjugating enzyme E2 26 | 8.27 |
|  | GRMZM2G449875 | NP_001150741 | RING-H2 finger protein ATL73 | 8.27 |
|  | GRMZM2G123922 |  | ATP-dependent Clp protease ATP-binding subunit clpA homolog CD4B, chloroplastic | 8.27 |
|  | GRMZM2G024312 | NP_001151920 | E3 ubiquitin-protein ligase ATL4 | 8.17 |
|  | GRMZM2G146745 | NM_001165787 | Cysteine proteinase RD19a | 5.75 |
|  | GRMZM2G181322 |  | Uncharacterized protein At5g39865 | 5.6 |
| Signal transduction mechanisms | GRMZM2G474755 | NP_001146874 | Probable calcium-binding protein CML15 | 12.17 |
|  | GRMZM2G019819 |  | Probable protein phosphatase 2C 24 | 10.01 |
|  | GRMZM2G086577 | NP_001136568 | Probable receptor-like serine/threonine-protein kinase At5g57670 | 9.79 |
|  | GRMZM2G428379 |  | Glutamate receptor 2.8 | 8.89 |
|  | GRMZM2G166658 |  | CBL-interacting protein kinase 2 | 8.69 |
|  | GRMZM2G102858 | NP_001150156 | Serine/threonine-protein phosphatase 2A 65 kDa regulatory subunit A beta isoform | 8.69 |
|  | GRMZM2G148807 |  | Glutamate receptor 3.4 | 8.37 |
|  | GRMZM2G311220 | NP_001149916 | Calcium-dependent protein kinase 13 | 8.37 |
|  | GRMZM2G158252 | NP_001104867 | Histidine kinase 2 | 8.17 |
|  | GRMZM2G332660 |  | Calcium-dependent protein kinase 13 | 7.95 |
|  | GRMZM2G176519 | NP_001147043 | CBL-interacting protein kinase 19 | 7.95 |
| Chromatin structure and dynamics | GRMZM2G143780 | P62787 | Histone H4 | 8.89 |
|  | GRMZM2G305046 | P40280 | Histone H2A | 5.62 |
| Replication, recombination and repair | GRMZM2G117028 | NP_001146598 | DNA polymerase III subunit gamma/tau | 9.45 |
|  | GRMZM2G394962 | NM_001148228 | Protein timeless homolog | 9.27 |
|  | GRMZM2G065804 |  | Ribonuclease H2 subunit A | 8.46 |
|  | GRMZM2G393742 |  | DNA repair protein rhp54 | 8.37 |
| RNA processing and modification | GRMZM2G076399 | NP_001147167 | Probable small nuclear ribonucleoprotein Sm D2 | 9.22 |
|  | GRMZM2G118104 | NP_001159123 | Cleavage and polyadenylation specificity factor subunit 5 | 8.61 |
|  | GRMZM2G161004 | NP_001130664 | Coiled-coil domain-containing protein 75 | 8.46 |
|  | GRMZM2G458401 |  | 5'-3' exoribonuclease 2 | 5.35 |
| Transcription | GRMZM2G124663 |  | Nuclear transcription factor Y subunit B-6 | 12.47 |
|  | GRMZM2G011789 | NP_001105518 | Nuclear transcription factor Y subunit B-6 | 11.54 |
|  | GRMZM2G165488 | NP_001131729 | Nuclear transcription factor Y subunit A-10 | 10.41 |
|  | GRMZM2G361659 | NP_001167717 | E2F transcription factor-like E2FE | 9.89 |
|  | GRMZM2G051528 | NP_001105937 | Transcription factor MYB12 | 9.76 |
|  | GRMZM2G135381 |  | GATA transcription factor 12 | 9.69 |
|  | GRMZM2G055158 | NM_001156302 | Protein ODORANT1 | 9.22 |
|  | GRMZM2G123202 | Q8S417 | Myb-related protein Myb4 | 9.17 |
|  | GRMZM2G088783 | NM_001154866 | Transcription factor MYB86 | 9.17 |
|  | GRMZM2G378665 |  | E2F transcription factor-like E2FE | 9.17 |
|  | GRMZM2G165011 | NP_001168654 | Probable histone-lysine N-methyltransferase, H3 lysine-9 specific SUVH9 | 8.54 |
| Translation, ribosomal structure and biogenesis | GRMZM2G065956 | NP_001150377 | 50S ribosomal protein L7/L12 | 8.61 |
|  | GRMZM2G053466 | NM_001147955 | Glutamyl-tRNA(Gln) amidotransferase subunit A | 8.07 |
|  | GRMZM2G116133 | NM_001153345 | Exosome complex component RRP42 | 7.95 |
|  | GRMZM2G156565 | C0PHB3 | Cysteinyl-tRNA synthetase | 5.12 |
| Amino acid transport and metabolism | GRMZM2G047404 | NP_001105952 | Phosphoenolpyruvate/phosphate translocator 1, chloroplastic | 8.83 |
|  | GRMZM2G124963 | NP_001145656 | Alanine aminotransferase 2 | 8.46 |
|  | GRMZM2G108125 | NP_001152508 | Uncharacterized aminotransferase y4uB | 8.37 |
|  | GRMZM2G058584 | NP_001159131 | Histidinol dehydrogenase, chloroplastic | 8.27 |
|  | GRMZM2G569855 |  | Threonine dehydratase biosynthetic, chloroplastic | 8.07 |
| Carbohydrate transport and metabolism | GRMZM2G039845 |  | Probable aquaporin TIP3-2 | 8.61 |
|  | GRMZM2G149841 |  | Probable beta-1,3-galactosyltransferase 19 | 8.37 |
|  | GRMZM2G063048 |  | Uncharacterized protein At2g34460, chloroplastic | 8.27 |
|  | GRMZM2G074946 | NP_001149351 | Probable 6-phosphogluconolactonase 1 | 7.95 |
|  | GRMZM2G141510 | NP_001149021 | Xylulose kinase | 7.95 |
|  | GRMZM2G376416 | C4J2I7 | Beta-glucosidase 31 | 7.95 |
|  | GRMZM2G103983 | Q9AT75 | Aquaporin TIP3-2 | 6.1 |
|  | GRMZM2G013255 | NP_001151568 | Alpha-L-fucosidase 1 | 5.12 |
| Energy production and conversion | GRMZM2G432291 |  | Cyanohydrin beta-glucosyltransferase | 10.69 |
|  | GRMZM2G428027 |  | Nitrate reductase [NAD(P)H] | 10.09 |
|  | GRMZM2G466833 | NP_001140825 | Malate dehydrogenase, mitochondrial | 9.17 |
|  | GRMZM2G117786 | NP_001145861 | Dihydrolipoyllysine-residue acetyltransferase component of pyruvate dehydrogenase complex | 8.37 |
|  | GRMZM2G469489 |  | NADH-ubiquinone oxidoreductase chain 2 | 8.07 |
|  | GRMZM2G395508 |  | Anthocyanidin 5,3-O-glucosyltransferase | 7.95 |
|  | GRMZM2G086925 | NP_001136576 | Cis-zeatin O-glucosyltransferase 1 | 7.95 |
|  | GRMZM2G174773 |  | Glyoxylate reductase | 7.95 |
|  | GRMZM2G033515 | NP_001136968 | Isocitrate dehydrogenase [NADP] | 5.5 |
|  | GRMZM2G127546 | NP_001148617 | Pyruvate dehydrogenase E1 component subunit beta | 5.06 |
| Inorganic ion transport and metabolism | GRMZM2G112377 | NP_001105817 | Inorganic phosphate transporter 1-6 | 14.19 |
|  | GRMZM2G370780 |  | SPX domain-containing protein 5 | 9.22 |
|  | GRMZM2G009368 |  | Ethylene-insensitive protein 2 | 8.61 |
|  | GRMZM2G148374 | NP_001169998 | ATPase 8, plasma membrane-type | 8.46 |
|  | GRMZM2G037343 |  | Cation/H(+) antiporter 15 | 8.27 |
|  | GRMZM2G065989 |  | SPX domain-containing protein 6 | 6.25 |
| Lipid transport and metabolism | GRMZM2G050658 | NP_001141145 | Lipid phosphate phosphatase 2 | 9.58 |
|  | GRMZM2G117064 |  | Long chain acyl-CoA synthetase 9, chloroplastic | 9.22 |
|  | GRMZM2G029543 | NP_001150334 | Malonyl-CoA-acyl carrier protein transacylase, mitochondrial | 8.76 |
|  | GRMZM2G066321 | NP_001152241 | Putative C-4 methylsterol oxidase DDB_G0269788 | 8.61 |
|  | GRMZM2G167673 |  | Secologanin synthase | 7.95 |
| Nucleotide transport and metabolism | GRMZM2G080387 |  | Nucleobase-ascorbate transporter 2 | 9.45 |
|  | GRMZM2G036120 |  | Epsin-2 | 8.69 |
| Secondary metabolites biosynthesis, transport and catabolism | GRMZM2G030831 | NP_001132060 | Cytochrome P450 89A2 | 11.12 |
|  | GRMZM2G034471 |  | Cytochrome P450 78A3 | 11.11 |
|  | GRMZM2G477872 |  | Pleiotropic drug resistance protein 2 | 9.27 |
|  | GRMZM2G147966 |  | Respiratory burst oxidase homolog protein E | 9.01 |
|  | GRMZM2G177314 | NP_001147125 | ABC transporter G family member 11 | 8.69 |
|  | GRMZM2G091819 | NP_001105991 | Putative flavin-containing monooxygenase YUCCA11 | 8.37 |
|  | GRMZM2G470442 |  | Cytochrome P450 78A3 | 8.26 |
|  | GRMZM2G359298 | NM_001175055 | Primary amine oxidase | 8.17 |
|  | GRMZM2G019866 | NP_001146847 | Acyl carrier protein 3, chloroplastic | 6 |
|  | GRMZM2G132450 | B6SZ21 | Cytochrome P450 81D1 | 5.62 |
