# Supplementary material for: Genome Expression Profile Analysis of the Immature Maize Embryo during Dedifferentiation
Source: PLoS One. 2012 Mar 20;7(3):e32237. doi: 10.1371/journal.pone.0032237 (PMC3308947; doi:10.1371/journal.pone.0032237)
Supplement: Table S5 — List of DEGs changed for at least 5 folds in stage III sample. (DOC) [file pone.0032237.s006.doc]

Table S5. List of DEGs changed for at least 5 folds in stage III sample.

| **Function classification** | **Gene** | **Accession** | **Function annotation** | **Fold** |
| --- | --- | --- | --- | --- |
| **Up-regulated genes** | | | | |
| Cell cycle control, cell division, chromosome partitioning | GRMZM2G096228 | NP_001149887 | Probable calcium-binding protein CML8 | -9.71 |
|  | GRMZM2G096691 |  | Mps one binder kinase activator-like 1 | -8.37 |
| Cell wall/membrane/envelope biogenesis | GRMZM2G447795 | NP_001151661 | Xylanase inhibitor protein 1 | -16.18 |
|  | GRMZM2G328171 | NP_001146870 | Xylanase inhibitor protein 1 | -12.14 |
|  | GRMZM2G072034 | NP_001140887 | Putative lipocalin R877 | -11.08 |
|  | GRMZM2G162359 | NP_001142312 | Xylanase inhibitor protein 1 | -10.79 |
|  | GRMZM2G130276 |  | Acidic endochitinase | -8.29 |
|  | GRMZM2G435120 |  | Galactomannan galactosyltransferase 1 | -8.29 |
|  | GRMZM2G133781 | NP_001140795 | Xylanase inhibitor protein 2 | -5.32 |
| Cytoskeleton | GRMZM2G175761 | NP_001148027 | Pollen-specific protein SF3 | -10.84 |
|  | GRMZM2G151934 |  | Protein DA1-related 2 | -10.09 |
|  | GRMZM2G429928 |  | Formin-like protein 4 | -8.81 |
|  | GRMZM2G132958 | NP_001144650 | IST1-like protein | -8.61 |
|  | GRMZM2G066191 | Q41782 | Tubulin beta-4 chain | -5.23 |
| Defense mechanisms | GRMZM2G099420 |  | Dihydroflavonol-4-reductase | -13.8 |
|  | GRMZM2G013726 | NP_001140905 | Dihydroflavonol-4-reductase | -9.64 |
|  | GRMZM2G301934 | NP_001150053 | Probable carboxylesterase 18 | -9.57 |
|  | GRMZM2G104141 |  | Probable carboxylesterase 18 | -8.74 |
|  | GRMZM2G131836 | NP_001105715 | Dihydroflavonol-4-reductase | -8.19 |
| Intracellular trafficking, secretion, and vesicular transport | GRMZM2G154735 | NP_001147407 | Protein HVA22 | -10.24 |
|  | GRMZM2G023436 | NP_001131720 | Ras-related protein RABA1f | -9.46 |
|  | GRMZM2G172834 | NP_001147343 | Annexin D5 | -9.04 |
|  | GRMZM2G003769 | NP_001146842 | Peroxisomal membrane protein 11-4 | -8.37 |
|  | GRMZM2G029270 |  | Syntaxin-124 | -7.87 |
|  | GRMZM2G061950 | NP_001105475 | Annexin D1 | -5.15 |
| Posttranslational modification, protein turnover, chaperones | GRMZM2G150276 | NP_001105571 | Oryzain alpha chain | -16.27 |
|  | GRMZM2G124684 | NP_001150335 | Aspartic proteinase nepenthesin-2 | -12.58 |
|  | GRMZM2G120079 | NP_001159301 | Metacaspase-1 | -12.57 |
|  | GRMZM2G448368 |  | Chaperone protein dnaJ 11, chloroplastic | -12.43 |
|  | GRMZM2G324956 | NM_001157527 | 18.9 kDa heat shock protein | -12.13 |
|  | GRMZM2G480106 | NP_001144642 | RING-H2 finger protein ATL32 | -11.83 |
|  | GRMZM2G118366 | NP_001145886 | Uncharacterized protein At5g39865 | -11.52 |
|  | GRMZM2G010491 | NP_001167771 | Thioredoxin H2-1 | -11.2 |
|  | GRMZM2G156877 | NP_001105720 | Glutathione S-transferase 4 | -11.07 |
|  | GRMZM2G120587 | NM_001153228 | Serine carboxypeptidase-like 51 | -10.91 |
|  | GRMZM2G022799 | NM_001152659 | Metacaspase-9 | -10.79 |
|  | GRMZM2G429396 | NP_001147410 | 18.8 kDa class V heat shock protein | -10.64 |
|  | GRMZM2G401848 | NP_001149128 | Peptidyl-prolyl cis-trans isomerase | -10.62 |
|  | GRMZM2G436084 | NP_001158966 | Thioredoxin H-type 2 | -10.28 |
|  | GRMZM2G053206 | NP_001140805 | Basic 7S globulin | -10.21 |
|  | GRMZM2G166870 | NP_001140922 | Cysteine proteinase EP-B 1 | -9.81 |
|  | GRMZM2G021796 | NP_001151934 | RING-H2 finger protein ATL2 | -9.78 |
|  | GRMZM2G014055 | NP_001147412 | Thioredoxin H-type | -9.74 |
|  | GRMZM2G028129 | NP_001130836 | Probable mitochondrial chaperone BCS1-B | -9.61 |
|  | GRMZM2G106165 | NP_001148149 | Serine carboxypeptidase-like 51 | -9.53 |
|  | GRMZM2G175593 | NP_001147824 | Probable mitochondrial chaperone bcs1 | -9.49 |
|  | GRMZM2G161827 |  | Probable glutathione S-transferase GSTU6 | -9.14 |
|  | GRMZM2G105523 | NP_001143732 | Pyrrolidone-carboxylate peptidase | -8.99 |
|  | GRMZM2G300589 | NP_001144032 | E3 ubiquitin-protein ligase RING1-like | -8.74 |
|  | GRMZM2G468657 | NP_001150678 | Aspartic proteinase nepenthesin-1 | -8.68 |
|  | GRMZM2G340416 |  | Wall-associated receptor kinase 3 | -8.68 |
|  | GRMZM2G096247 | NP_001148830 | Glutathione S-transferase 6, chloroplastic | -8.53 |
|  | GRMZM2G118646 | NP_001149027 | Protease Do-like 5, chloroplastic | -8.53 |
|  | GRMZM2G025954 | NP_001140943 | Thioredoxin F, chloroplastic | -8.53 |
|  | GRMZM2G098102 | NP_001130503 | Cysteine proteinase EP-B 1 | -8.46 |
|  | GRMZM2G161891 | NP_001105511 | Probable glutathione S-transferase GSTU6 | -8.29 |
|  | GRMZM2G042639 | P49248 | Protein IN2-1 | -8.29 |
|  | GRMZM2G367701 | NP_001150119 | Xylem cysteine proteinase 2 | -8.09 |
|  | GRMZM2G144197 | NP_001150338 | Probable mitochondrial chaperone bcs1 | -7.98 |
|  | GRMZM2G097856 |  | 14-3-3-like protein GF14-D | -7.87 |
|  | GRMZM2G428179 | NP_001148308 | RING-H2 finger protein ATL40 | -7.87 |
|  | GRMZM2G025190 | B6T033 | Probable glutathione S-transferase GSTU6 | -7.53 |
|  | GRMZM2G040515 | NM_001154835 | Pyrrolidone-carboxylate peptidase | -6.58 |
|  | GRMZM2G073465 | NP_001149658 | Oryzain alpha chain | -6.2 |
|  | GRMZM2G035045 | NP_001140873 | Cysteine proteinase EP-B 2 | -6.09 |
|  | GRMZM2G434541 |  | Probable glutathione S-transferase GSTU6 | -5.75 |
|  | GRMZM2G040803 | NP_001152715 | E3 ubiquitin-protein ligase RMA1H1 | -5.68 |
|  | GRMZM2G323757 |  | Basic 7S globulin | -5.49 |
|  | GRMZM2G066326 | NP_001149806 | Xylem cysteine proteinase 2 | -5.41 |
|  | GRMZM2G103628 | NP_001105786 | Probable glucuronosyltransferase Os05g0559600 | -5.26 |
|  | GRMZM2G132509 | NP_001142024 | Basic 7S globulin | -5.03 |
| Signal transduction mechanisms | GRMZM2G043799 | NP_001151810 | Probable leucine-rich repeat receptor-like protein kinase At4g00330 | -10.93 |
|  | GRMZM2G459663 | NP_001152636 | Probable calcium-binding protein CML45 | -10.79 |
|  | GRMZM2G066432 | NP_001148201 | Serine/threonine-protein kinase At3g07070 | -10.79 |
|  | GRMZM2G359986 | NP_001168336 | Wall-associated receptor kinase-like 20 | -10.55 |
|  | GRMZM2G062673 | NP_001147205 | Probable calcium-binding protein CML22 | -10.43 |
|  | GRMZM2G401664 | B4FUX3 | 1-phosphatidylinositol phosphodiesterase | -10.28 |
|  | GRMZM2G050959 |  | Disease resistance protein RPM1 | -10.04 |
|  | GRMZM2G330049 | NP_001152211 | CBL-interacting protein kinase 16 | -9.96 |
|  | GRMZM2G141975 | NM_001165629 | Oligopeptide transporter 4 | -9.64 |
|  | GRMZM2G066202 | NP_001152288 | Auxin-induced in root cultures protein 12 | -9.37 |
|  | GRMZM2G390896 | NP_001105966 | CBL-interacting protein kinase 5 | -9.24 |
|  | GRMZM2G028568 | NP_001170444 | Probable serine/threonine-protein kinase At1g18390 | -9.19 |
|  | GRMZM2G025579 |  | Histidine kinase 5 | -9.14 |
|  | GRMZM2G138355 | NP_001151118 | Nudix hydrolase 13, mitochondrial | -8.93 |
|  | GRMZM2G005557 |  | Probable calcium-binding protein CML9 | -8.87 |
|  | GRMZM2G053833 | NP_001148333 | Probable calcium-binding protein CML45 | -8.68 |
|  | GRMZM2G159908 | NP_001131825 | Putative serine/threonine-protein kinase-like protein CCR3 | -8.68 |
|  | GRMZM2G038893 | NP_001147438 | Wall-associated receptor kinase-like 14 | -8.37 |
|  | GRMZM2G156255 |  | Potassium channel SKOR | -8.29 |
|  | GRMZM2G161380 | NP_001169946 | Serine/threonine-protein kinase-like protein ACR4 | -8.29 |
|  | GRMZM2G110968 | NP_001140459 | Serine/threonine-protein kinase At5g01020 | -7.98 |
|  | GRMZM2G157384 |  | Rhomboid family member 1 | -7.87 |
|  | GRMZM2G147373 | NP_001141346 | Serine/threonine-protein kinase At5g01020 | -7.87 |
|  | GRMZM2G381071 | NP_001142366 | Polcalcin Phl p 7 | -7.07 |
| Chromatin structure and dynamics | GRMZM2G170201 | B6UI28 | Transcriptional regulatory protein SIN3 | -9.33 |
| RNA processing and modification | GRMZM2G141322 | NP_001151299 | Ribonuclease 1 | -11.61 |
|  | GRMZM2G322506 |  | Helicase SEN1 | -8.61 |
|  | GRMZM2G404375 | NM_001174609 | Ribonuclease J | -8.09 |
| Transcription | GRMZM2G041462 | C4J8W1 | Homeobox-leucine zipper protein HOX6 | -11.46 |
|  | GRMZM2G139535 | NP_001150318 | Heat stress transcription factor B-1 | -11.43 |
|  | GRMZM2G095598 |  | Zinc finger protein CONSTANS-LIKE 3 | -10.82 |
|  | GRMZM2G134260 |  | Homeobox-leucine zipper protein HOX2 | -10.43 |
|  | GRMZM2G050305 | NP_001105949 | Myb-related protein Hv1 | -9.53 |
|  | GRMZM2G139073 | NP_001104951 | MADS-box transcription factor 16 | -9.29 |
|  | GRMZM2G458728 |  | BEL1-like homeodomain protein 1 | -9.14 |
|  | GRMZM2G021339 | NP_001169496 | Homeobox-leucine zipper protein HOX16 | -8.93 |
|  | GRMZM2G004641 | NP_001146740 | BEL1-like homeodomain protein 7 | -8.87 |
|  | GRMZM2G099319 | NP_001131173 | BEL1-like homeodomain protein 6 | -8.68 |
|  | GRMZM2G034113 | NP_001149510 | Homeobox-leucine zipper protein HOX6 | -8.68 |
|  | GRMZM2G305856 | Q9S7U6 | Myb-related protein MYBAS2 | -8.68 |
|  | GRMZM2G147716 | NP_001105155 | MADS-box transcription factor 18 | -8.29 |
|  | GRMZM2G159431 | P56659 | Homeobox protein knotted-1-like 3 | -8.19 |
|  | GRMZM2G047600 |  | Myb-related protein 305 | -8.19 |
|  | GRMZM2G022162 |  | Nuclear transcription factor Y subunit C-2 | -7.98 |
|  | GRMZM2G156348 | NP_001149390 | Trihelix transcription factor GT-3b | -7.98 |
|  | GRMZM2G137046 | NP_001152483 | Transcription factor HY5 | -7.87 |
|  | GRMZM2G002131 |  | Heat stress transcription factor B-1 | -6.15 |
|  | GRMZM2G148772 |  | Zinc finger protein CONSTANS-LIKE 3 | -5.98 |
|  | GRMZM2G106276 | NP_001150756 | Homeobox-leucine zipper protein HOX28 | -5.8 |
|  | GRMZM2G044576 | NP_001159272 | GATA transcription factor 12 | -5.18 |
| Translation, ribosomal structure and biogenesis | GRMZM2G542753 |  | 40S ribosomal protein S4 | -8.19 |
| Amino acid transport and metabolism | GRMZM2G085381 | P42390 | Indole-3-glycerol phosphate lyase, chloroplastic | -13.48 |
|  | GRMZM2G048434 | NP_001136826 | Uncharacterized membrane protein At1g06890 | -11.95 |
|  | GRMZM2G042933 | NP_001137042 | Amino acid permease 2 | -11.26 |
|  | GRMZM2G061303 |  | Nitrate transporter 1.5 | -11.14 |
|  | GRMZM2G178734 |  | Probable sugar phosphate/phosphate translocator At5g25400 | -10.57 |
|  | GRMZM2G089140 |  | Probable nitrite transporter At1g68570 | -10.39 |
|  | GRMZM2G433767 | NP_001147904 | Serine carboxypeptidase II-3 | -10.35 |
|  | GRMZM2G046601 | NM_001111827 | Glutamine synthetase root isozyme 5 | -9.87 |
|  | GRMZM2G126541 |  | Serine carboxypeptidase-like 50 | -9.84 |
|  | GRMZM2G125923 | NP_001146088 | Arogenate dehydratase/prephenate dehydratase 6, chloroplastic | -9.71 |
|  | GRMZM2G009400 | NP_001147982 | Tyrosine/DOPA decarboxylase 2 | -9.64 |
|  | GRMZM2G127328 | NP_001147827 | Lysine histidine transporter 2 | -9.46 |
|  | GRMZM2G161696 | NP_001152245 | Serine carboxypeptidase-like 34 | -9.29 |
|  | GRMZM2G161641 |  | Amino acid permease 3 | -8.99 |
|  | GRMZM2G044851 | NM_001177082 | Nitrate transporter 1.5 | -8.81 |
|  | GRMZM2G069203 | NP_001105082 | Probable serine acetyltransferase 1 | -8.74 |
|  | GRMZM2G001676 |  | Peptide transporter PTR3-A | -8.61 |
|  | GRMZM2G395236 |  | Dihydrofolate reductase | -8.37 |
|  | GRMZM2G122712 |  | Probable nitrite transporter At1g68570 | -8.37 |
|  | GRMZM2G116554 | NP_001146361 | Uncharacterized amino-acid permease C15C4.04c | -8.37 |
|  | GRMZM2G154958 |  | Lysine histidine transporter 1 | -8.19 |
|  | GRMZM2G112039 |  | Probable peptide/nitrate transporter At1g59740 | -8.19 |
|  | GRMZM2G015892 | B4F800 | Indole-3-glycerol phosphate lyase, chloroplastic | -7.98 |
|  | GRMZM2G052288 |  | Ornithine decarboxylase | -7.98 |
|  | GRMZM2G111164 | NP_001132769 | Probable bifunctional methylthioribulose-1-phosphate dehydratase/enolase-phosphatase E1 | -7.87 |
|  | GRMZM2G036708 | NP_001136599 | Cysteine synthase, chloroplastic/chromoplastic | -7.28 |
|  | GRMZM2G098875 | NP_001167941 | Glutamate decarboxylase 1 | -6.47 |
|  | GRMZM2G327595 | NP_001147829 | Serine carboxypeptidase II-3 | -6.21 |
| Carbohydrate transport and metabolism | GRMZM2G016890 | P49235 | Beta-glucosidase, chloroplastic | -15.63 |
|  | GRMZM2G008247 | NP_001105892 | Beta-glucosidase, chloroplastic | -13.94 |
|  | GRMZM2G081843 | Q9AR14 | Aquaporin PIP1-5 | -13.62 |
|  | GRMZM2G120962 |  | Beta-glucosidase, chloroplastic | -13.05 |
|  | GRMZM2G115124 | NP_001149057 | GDP-mannose 4,6 dehydratase 2 | -12.82 |
|  | GRMZM2G138468 | NP_001105539 | Alpha-amylase isozyme 3B | -12.08 |
|  | GRMZM2G007263 |  | Glyceraldehyde-3-phosphate dehydrogenase B, chloroplastic | -11.18 |
|  | GRMZM2G081192 | Q9ATM7 | Aquaporin PIP2-3 | -10.57 |
|  | GRMZM2G055699 | NP_001145839 | Beta-glucosidase 22 | -10.01 |
|  | GRMZM2G178693 | Q9XF58 | Aquaporin PIP2-5 | -9.99 |
|  | GRMZM2G401970 |  | 6-phosphofructokinase 3 | -9.09 |
|  | GRMZM2G125023 | Q84RL6 | Aquaporin TIP2-3 | -8.93 |
|  | GRMZM2G108133 | C4JAJ7 | Beta-glucosidase 31 | -8.74 |
|  | GRMZM2G031169 | NP_001151520 | Uncharacterized protein At2g34460, chloroplastic | -8.68 |
|  | GRMZM2G082184 | NP_001131324 | Aquaporin NIP2-1 | -8.29 |
|  | GRMZM2G118378 |  | Putative protein PLEKHA9 | -8.09 |
|  | GRMZM2G048230 |  | Trehalose-phosphate phosphatase | -8.09 |
|  | GRMZM2G154628 | Q9ATM6 | Aquaporin PIP2-4 | -7.26 |
|  | GRMZM2G041356 | NP_001137118 | Aldose 1-epimerase | -6.14 |
|  | GRMZM2G027098 | Q9ATL9 | Aquaporin TIP2-1 | -5.91 |
|  | GRMZM2G062201 |  | Probable inactive purple acid phosphatase 1 | -5.55 |
|  | GRMZM2G142919 | NP_001148061 | Probable anion transporter 2, chloroplastic | -5.41 |
| Coenzyme transport and metabolism | GRMZM2G038821 | Q05326 | Pyruvate decarboxylase isozyme 1 | -5.41 |
| Energy production and conversion | GRMZM2G168474 | Q93XP7 | Cis-zeatin O-glucosyltransferase 1 | -12.81 |
|  | GRMZM2G159724 | NP_001152396 | NADP-dependent malic enzyme | -12.64 |
|  | GRMZM2G118800 | NP_001168661 | Aldehyde dehydrogenase family 3 member H1 | -12.42 |
|  | GRMZM2G144081 | NM_001112419 | Protein brittle-1, chloroplastic/amyloplastic | -11.4 |
|  | GRMZM2G041699 | NP_001149205 | Cytokinin-O-glucosyltransferase 2 | -10.19 |
|  | GRMZM2G130119 |  | Anthocyanidin 5,3-O-glucosyltransferase | -10.01 |
|  | GRMZM2G167220 | NP_001105163 | Cytokinin dehydrogenase 4 | -9.96 |
|  | GRMZM2G009045 |  | Phosphate carrier protein, mitochondrial | -9.78 |
|  | GRMZM2G078465 | NP_001150551 | Indole-3-acetate beta-glucosyltransferase | -9.74 |
|  | GRMZM2G399338 |  | Transcription factor GTE8 | -9.49 |
|  | GRMZM2G059129 |  | Probable glycerophosphoryl diester phosphodiesterase 1 | -9.46 |
|  | GRMZM2G098890 | NP_001170719 | Cytokinin-O-glucosyltransferase 2 | -9.33 |
|  | GRMZM2G169458 | NP_001147083 | Aldehyde dehydrogenase family 3 member F1 | -9.29 |
|  | GRMZM2G179063 | NP_001168657 | Flavonol-3-O-glycoside-7-O-glucosyltransferase 1 | -9.29 |
|  | GRMZM2G316030 |  | Indole-3-acetate beta-glucosyltransferase 1 | -8.93 |
|  | GRMZM2G049798 | NP_001168355 | Cytokinin-O-glucosyltransferase 2 | -8.81 |
|  | GRMZM2G061321 | NP_001150609 | Anthocyanidin 3-O-glucosyltransferase | -8.61 |
|  | GRMZM2G046994 |  | Cytokinin-O-glucosyltransferase 2 | -7.98 |
|  | GRMZM2G173192 | NP_001131896 | L-lactate dehydrogenase A | -7.87 |
|  | GRMZM2G010987 | NP_001149462 | Anthocyanidin 5,3-O-glucosyltransferase | -5.68 |
|  | GRMZM2G142386 |  | Nitrate reductase [NADH] 1 | -5.41 |
|  | GRMZM2G173536 | C0P676 | SNF1-related protein kinase regulatory subunit gamma 1 | -5 |
| Inorganic ion transport and metabolism | GRMZM2G102959 | NM_001177189 | Ferredoxin--nitrite reductase, chloroplastic (Fragment) | -12.41 |
|  | GRMZM2G090568 | P12365 | Catalase isozyme 2 | -12.39 |
|  | GRMZM2G086066 | NP_001152036 | Superoxide dismutase 1 copper chaperone | -12.31 |
|  | GRMZM2G344163 | NP_001169702 | Putative chloride channel-like protein CLC-g | -11.14 |
|  | GRMZM2G175140 | NP_001140828 | Ammonium transporter 1 member 1 | -10.37 |
|  | GRMZM2G047762 |  | Zinc transporter 5 | -10.14 |
|  | GRMZM2G057616 | C9DQ40 | Chloride channel protein CLC-a | -9.93 |
|  | GRMZM2G151406 |  | Copper-transporting ATPase RAN1 | -9.14 |
|  | GRMZM2G046480 |  | Vacuolar cation/proton exchanger 2 | -9.04 |
|  | GRMZM2G115190 |  | Fe(2+) transport protein 2 | -8.74 |
|  | GRMZM2G161846 | NP_001168502 | Superoxide dismutase 1 copper chaperone | -8.37 |
|  | GRMZM2G079381 | P17847 | Ferredoxin--nitrite reductase, chloroplastic (Fragment) | -8.09 |
|  | GRMZM2G080045 | NP_001145797 | Ammonium transporter 2 member 1 | -7.98 |
|  | GRMZM2G080178 | NP_001132356 | Sulfate transporter 1.3 | -7.87 |
|  | GRMZM2G096365 | NP_001170245 | Chloride channel protein CLC-c | -5.34 |
|  | GRMZM2G079348 | P18123 | Catalase isozyme 3 | -5.19 |
| Lipid transport and metabolism | GRMZM2G179147 | NP_001169802 | Abscisic acid 8'-hydroxylase 1 | -12.69 |
|  | GRMZM2G154523 | NP_001151190 | Patatin group A-3 | -12.24 |
|  | GRMZM2G150907 | NP_001140247 | Secologanin synthase | -10.64 |
|  | GRMZM2G032896 | B6T0I8 | Cytochrome P450 90D2 | -10.39 |
|  | GRMZM2G057136 |  | Triacylglycerol lipase 2 | -10.28 |
|  | GRMZM2G061969 | NP_001146559 | Phospholipase D alpha 1 | -10.26 |
|  | GRMZM2G363429 | C4J505 | Cytochrome P450 26C1 | -10.09 |
|  | GRMZM2G002142 |  | Abscisic acid 8'-hydroxylase 3 | -10.01 |
|  | GRMZM2G164074 | NP_001168221 | Cytochrome P450 94A1 | -9.33 |
|  | GRMZM2G370745 | NP_001141098 | Secologanin synthase | -9.24 |
|  | GRMZM2G024144 |  | Lipid phosphate phosphatase 2 | -8.37 |
|  | GRMZM2G481362 |  | Acyl-protein thioesterase 1 | -8.09 |
|  | GRMZM2G446454 | NM_001147318 | Cytochrome P450 87A3 | -8.09 |
|  | GRMZM2G176943 |  | Secologanin synthase | -8.09 |
|  | GRMZM2G126083 |  | Monoglyceride lipase | -7.98 |
|  | GRMZM2G172098 | NP_001136742 | Monoglyceride lipase | -7.87 |
|  | GRMZM2G048522 | NP_001130893 | Probable 4-coumarate--CoA ligase 5 | -7.87 |
|  | GRMZM2G396248 |  | Cytochrome P450 94A1 | -7.14 |
| Secondary metabolites biosynthesis, transport and catabolism | GRMZM2G085661 | Q43257 | Cytochrome P450 71C4 | -12.72 |
|  | GRMZM2G354909 | NP_001146814 | (+)-neomenthol dehydrogenase | -11.5 |
|  | GRMZM2G170692 | NP_001168086 | Phenylalanine ammonia-lyase | -11.41 |
|  | GRMZM2G152975 | NP_001169684 | Alcohol dehydrogenase-like 4 | -11.22 |
|  | GRMZM2G170017 | NP_001147467 | Salutaridine reductase | -10.85 |
|  | GRMZM2G312069 | NP_001142304 | Isoflavone 2'-hydroxylase | -10.19 |
|  | GRMZM2G432480 | NP_001152673 | Protein STAR1 | -10.19 |
|  | GRMZM2G154870 |  | Isoflavone 2'-hydroxylase | -10.04 |
|  | GRMZM2G025860 | NP_001169993 | Putative ABC transporter B family member 8 | -9.57 |
|  | GRMZM2G148052 |  | Cytochrome P450 71D7 | -9.53 |
|  | GRMZM2G063917 | NP_001147922 | Phenylalanine ammonia-lyase | -9.37 |
|  | GRMZM2G087875 | NP_001146006 | Isoflavone 2'-hydroxylase | -8.99 |
|  | GRMZM2G113844 | B4FUC5 | Probable flavin-containing monooxygenase 1 | -8.81 |
|  | GRMZM2G121264 | NP_001169622 | Cytochrome P450 71C4 | -8.37 |
|  | GRMZM2G154828 | NP_001130688 | Isoflavone 2'-hydroxylase | -7.98 |
|  | GRMZM2G110192 |  | Probable carotenoid cleavage dioxygenase 4, chloroplastic | -7.98 |
|  | GRMZM2G033952 | NP_001152511 | Probable caffeoyl-CoA O-methyltransferase At4g34050 | -7.87 |
|  | GRMZM2G160541 |  | Phenylalanine ammonia-lyase | -6.43 |
|  | GRMZM2G028677 | NP_001151365 | Trans-cinnamate 4-monooxygenase | -6.07 |
|  | GRMZM2G074604 | NP_001105334 | Phenylalanine ammonia-lyase | -5.63 |
|  | GRMZM2G167549 | Q43255 | Cytochrome P450 71C2 | -5.01 |
| **Down-regulated genes** | | | | |
| Cell cycle control, cell division, chromosome partitioning | GRMZM2G056303 | NP_001169350 | Cyclin-D5-1 | 7.95 |
| Cytoskeleton | GRMZM2G417410 |  | Formin-like protein 12 | 8.69 |
|  | GRMZM2G471108 |  | Myosin-J heavy chain | 8.37 |
| Defense mechanisms | GRMZM2G112792 |  | L-gulonolactone oxidase | 8.46 |
|  | GRMZM2G026095 |  | Probable carboxylesterase 12 | 8.17 |
|  | GRMZM2G391795 | C4J9M8 | Probable carboxylesterase 15 | 5.05 |
| Intracellular trafficking, secretion, and vesicular transport | GRMZM2G429781 |  | E3 ubiquitin-protein ligase HOS1 | 7.83 |
| Posttranslational modification, protein turnover, chaperones | GRMZM2G071272 |  | Probable mitochondrial chaperone bcs1 | 10.69 |
|  | GRMZM2G346839 | NP_001149613 | 23.2 kDa heat shock protein | 9.27 |
|  | GRMZM2G157855 | NP_001152117 | E3 ubiquitin-protein ligase RING1-like | 8.69 |
|  | GRMZM2G063192 | NP_001151581 | Anaphase-promoting complex subunit cdc20 | 8.37 |
|  | GRMZM2G024312 | NP_001151920 | E3 ubiquitin-protein ligase ATL4 | 8.17 |
|  | GRMZM2G049767 | NP_001168642 | 16.6 kDa heat shock protein | 5.03 |
| Signal transduction mechanisms | GRMZM2G428379 |  | Glutamate receptor 2.8 | 8.89 |
|  | GRMZM2G102858 | NP_001150156 | Serine/threonine-protein phosphatase 2A 65 kDa regulatory subunit A beta isoform | 8.69 |
|  | GRMZM2G142068 |  | Proline-rich receptor-like protein kinase PERK9 | 8.07 |
|  | GRMZM2G054152 | NM_001174621 | Casein kinase I isoform delta-like | 8.07 |
|  | GRMZM2G332660 |  | Calcium-dependent protein kinase 13 | 7.95 |
|  | GRMZM2G176519 | NP_001147043 | CBL-interacting protein kinase 19 | 7.95 |
|  | GRMZM2G062761 | NP_001130402 | Mitogen-activated protein kinase 17 | 7.83 |
| Chromatin structure and dynamics | GRMZM2G442555 |  | Histone H2B.2 | 5.55 |
| Replication, recombination and repair | GRMZM2G393742 |  | DNA repair protein rhp54 | 8.37 |
| RNA processing and modification | GRMZM2G458401 |  | 5'-3' exoribonuclease 2 | 10.71 |
|  | GRMZM2G340618 |  | Helicase SEN1 | 7.95 |
|  | GRMZM2G144890 | NP_001141754 | RNA-binding protein Musashi homolog Rbp6 | 7.83 |
| Transcription | GRMZM2G124663 |  | Nuclear transcription factor Y subunit B-6 | 12.47 |
|  | GRMZM2G011789 | NP_001105518 | Nuclear transcription factor Y subunit B-6 | 11.54 |
|  | GRMZM2G055158 | NM_001156302 | Protein ODORANT1 | 9.22 |
|  | GRMZM2G123202 | Q8S417 | Myb-related protein Myb4 | 9.17 |
|  | GRMZM2G088783 | NM_001154866 | Transcription factor MYB86 | 9.17 |
|  | GRMZM2G179346 | NP_001149597 | DNA-directed RNA polymerase II subunit RPB7 | 8.07 |
|  | GRMZM2G163335 |  | Agamous-like MADS-box protein AGL61 | 7.95 |
| Translation, ribosomal structure and biogenesis | GRMZM2G141818 | NP_001169445 | Protein argonaute 4B | 9.5 |
|  | GRMZM2G065956 | NP_001150377 | 50S ribosomal protein L7/L12 | 8.61 |
|  | GRMZM2G116133 | NM_001153345 | Exosome complex component RRP42 | 7.95 |
| Amino acid transport and metabolism | GRMZM2G001764 | NP_001130733 | Peptide transporter PTR3-A | 11.09 |
|  | GRMZM2G018353 |  | Probable peptide/nitrate transporter At1g27040 | 7.83 |
| Carbohydrate transport and metabolism | GRMZM2G074946 | NP_001149351 | Probable 6-phosphogluconolactonase 1 | 7.95 |
|  | GRMZM2G376416 | C4J2I7 | Beta-glucosidase 31 | 7.95 |
|  | GRMZM2G051004 | NP_001130796 | Glyceraldehyde-3-phosphate dehydrogenase, cytosolic | 7.83 |
| Coenzyme transport and metabolism | GRMZM2G161673 | NP_001141335 | Magnesium-protoporphyrin O-methyltransferase | 9.07 |
| Energy production and conversion | GRMZM2G432291 |  | Cyanohydrin beta-glucosyltransferase | 10.69 |
|  | GRMZM2G428027 |  | Nitrate reductase [NAD(P)H] | 10.09 |
|  | GRMZM2G174773 |  | Glyoxylate reductase | 7.95 |
| Inorganic ion transport and metabolism | GRMZM2G370780 |  | SPX domain-containing protein 5 | 9.22 |
|  | GRMZM2G009368 |  | Ethylene-insensitive protein 2 | 8.61 |
|  | GRMZM2G148374 | NP_001169998 | ATPase 8, plasma membrane-type | 8.46 |
|  | GRMZM2G410990 |  | ATPase 6, plasma membrane-type | 8.46 |
|  | GRMZM2G037343 |  | Cation/H(+) antiporter 15 | 8.27 |
|  | GRMZM2G112377 | NP_001105817 | Inorganic phosphate transporter 1-6 | 7.59 |
|  | GRMZM2G065989 |  | SPX domain-containing protein 6 | 5.98 |
| Lipid transport and metabolism | GRMZM2G117064 |  | Long chain acyl-CoA synthetase 9, chloroplastic | 9.22 |
|  | GRMZM2G066321 | NP_001152241 | Putative C-4 methylsterol oxidase DDB_G0269788 | 8.61 |
|  | GRMZM2G014981 | NP_001151948 | Probable 1-acyl-sn-glycerol-3-phosphate acyltransferase 5 | 8.17 |
|  | GRMZM2G034534 | NM_001152951 | Lecithin-cholesterol acyltransferase-like 1 | 7.83 |
| Nucleotide transport and metabolism | GRMZM2G080387 |  | Nucleobase-ascorbate transporter 2 | 9.45 |
|  | GRMZM2G036120 |  | Epsin-2 | 8.69 |
| Secondary metabolites biosynthesis, transport and catabolism | GRMZM2G417954 | NP_001147781 | 9-cis-epoxycarotenoid dioxygenase 1, chloroplastic | 9.07 |
|  | GRMZM2G091819 | NP_001105991 | Putative flavin-containing monooxygenase YUCCA11 | 8.37 |
|  | GRMZM2G359298 | NM_001175055 | Primary amine oxidase | 8.17 |
|  | GRMZM2G019866 | NP_001146847 | Acyl carrier protein 3, chloroplastic | 6.07 |
|  | GRMZM2G030831 | NP_001132060 | Cytochrome P450 89A2 | 5.26 |
|  | GRMZM2G132450 | B6SZ21 | Cytochrome P450 81D1 | 5.12 |
